# Design optimization and real-time implementation of an LSPMSM for efficiency enhancement

**DOI:** 10.1038/s41598-025-21896-5

**Published:** 2025-10-30

**Authors:** Cemil Ocak, Burak Yenipınar, Emre Çelik, Mahmoud Abdel-Salam, Ghanshyam G. Tejani, Seyed Jalaleddin Mousavirad

**Affiliations:** 1 Department of Electrical and Electronics Engineering, Technology Faculty, Gazi University, Ankara, Türkiye; 2 Department of Electronic Technology, Vocational School, Ostim Technical University, Ankara, Türkiye; 3 Department of Electrical and Electronics Engineering, Engineering Faculty, Düzce University, Düzce, Türkiye; 4 Faculty of Computer and Information Science, Mansoura University, Mansoura, 35516 Egypt; 5 Department of Research Analytics, Saveetha Dental College and Hospitals, Saveetha Institute of Medical and Technical Sciences, Saveetha University, Chennai, 600077 India; 6 Department of Industrial Engineering and Management, Yuan Ze University, Taoyuan, 320315 Taiwan; 7 Department of Computer and Electrical Engineering, Mid Sweden University, Sundsvall, Sweden

**Keywords:** LSPMSM, IE4 efficiency, Optimization, FEM, Synchronization, Demagnetization, Payback, Electrical and electronic engineering, Computational science

## Abstract

This study presents the design optimization and experimental validation of a Line-Start Permanent Magnet Synchronous Motor (LSPMSM) aimed at achieving IE4 efficiency class. An IE1 class induction motor (IM) was used as a reference. Only the rotor structure was modified, while the stator geometry, winding, and mechanical components were kept unchanged. The optimization process focused on rotor slot geometry, magnet placement, magnet dimensions, and core length, employing a Multi-Objective Genetic Algorithm (MOGA) to maximize efficiency while maintaining cost-effectiveness. Following the optimization, six candidate designs were evaluated based on demagnetization prediction, synchronization performance, and starting torque capability. Among them, Design C demonstrated the highest overall performance. Finite Element Analysis (FEA) confirmed that Design C met IE4 efficiency standards with a calculated efficiency of 92.15%. This result was later experimentally verified at 91.95% through thermal testing. The study further examined the cost and payback period scenarios for adopting LSPMSMs in industrial applications. Three implementation strategies were analyzed: replacing only the rotor, purchasing a new IE4 LSPMSM instead of an IE1 motor, and replacing an operational IE1 motor with an IE4 LSPMSM. The results indicated that efficiency improvement could be achieved with minimal modifications. The payback period varied depending on the investment strategy. The findings demonstrate that high-efficiency LSPMSMs can serve as direct replacements for induction motors, offering energy savings and improved performance while maintaining compatibility with existing motor housings and components.

## Introduction

On June 6, 2005, the EC 32/2005 “Eco-design Directive”^1^ established a general framework for energy-consuming products. Subsequently, on July 22, 2009, the European Parliament issued the EC 640/2009 directive^2^, which defined the efficiency thresholds and the implementation timelines for electric motors. This directive mandated that, as of June 16, 2011, electric motors sold in the EU had to meet a minimum efficiency class of IE2. Furthermore, as of January 1, 2015, electric motors operating at mains voltage in the power range of 7.5–375 kW within European Union countries were required to meet a minimum efficiency class of IE3. At the same time, for electric motors used with variable speed drives, the minimum required efficiency class was specified as IE2. As of January 2017, while the efficiency limits remained unchanged, the applicable power range was expanded from 7.5 to 375 kW to 0.75–375 kW. With the European Union directive EU 2019/1781^3^, effective from July 1, 2021, electric motors in the 0.75–1000 kW range, whether operating at mains voltage or used with variable speed drives, were required to meet at least IE3 efficiency. Additionally, from this date onward, 2, 4, 6, and 8-pole motors within the power range of 0.12–0.75 kW were required to have a minimum efficiency class of IE2. In the second phase, effective from July 1, 2023, electric motors in the 75–200 kW range were mandated to meet a minimum efficiency class of IE4.

With increasingly stringent efficiency class requirements imposed by regulations, manufacturers are compelled to produce high-efficiency motors. However, a significant number of motors currently in use still belong to the IE1 and IE2 efficiency classes. To improve overall energy efficiency in the industry, these lower-efficiency motors need to be replaced with IE3 or IE4 equivalents. Nevertheless, when users opt to replace their existing motors with new high-efficiency models, the investment payback period tends to be long. This often fails to provide sufficient incentive for many users. Therefore, upgrading the efficiency class of existing low-efficiency motors at significantly lower cost is considered a key enabler for accelerating the transition to more efficient motor systems. It is evident that minimum efficiency requirements for industrial electric motors are progressively increasing worldwide, particularly in the European Union. As motors in the 75–200 kW range are now required to meet higher efficiency standards, future regulations are also expected to mandate IE4 levels for lower-power motors. Achieving IE4 efficiency in low-power motors poses several challenging design requirements. In this context, Line-Start Permanent Magnet Synchronous Motors (LSPMSMs) stand out in terms of efficiency compared to their asynchronous counterparts, offering IE4 efficiency even at lower power levels.

In this study, the efficiency values required for a 4-pole, 5.5 kW electric motor operating at 50 Hz mains voltage, optimized through design optimization, are presented in Table 1 according to different efficiency classes.

**Table 1:** Nominal efficiency limits for 1500 1/min 5.5 kW motor^4^.

IE1	IE2	IE3	IE4
84.7%	87.7%	89.6%	91.9%

Hyunwoo Kim et al.^5^ improved the efficiency and power factor of an LS-SynRM motor intended for industrial applications. The optimization was performed using the Response Surface Method (RSM) and finite element analysis (FEA) to meet the IE4 efficiency class requirements. Their analysis investigated the effects of barrier number and thickness, rotor bar depth, and the distance of rotor bars from the inner and outer rotor diameters (ribs) on both efficiency and power factor. As a result of their study, the optimized motor design achieved an efficiency of 91.7% and a power factor of 0.812, as confirmed by laboratory tests. Solmaz Kahourzade et al.^6^ designed, optimized, and tested a 3-phase, 4-pole, 2 kW LSPMSM by building a prototype. During the analysis phase, the transient and steady-state performance of the motor was evaluated using D-Q modeling and FEA. In the study, an improved design was achieved by optimizing the rotor bar geometry, magnet type, and magnet position within the rotor to enhance the starting and synchronization performance. Albert Johan Sorgdrager et al.^7^ developed an LSPMSM using the Taguchi method. Based on their experimental study, they proposed the use of the Taguchi Method-Based Regression Rate (TBRR) approach. This method addresses multi-objective optimization problems for LSPMSMs by considering both transient and steady-state performance. Abdul Waheed et al.^8^ developed an optimized motor model to improve the efficiency and power factor of an LSPMSM through parametric analyses based on magnetic equivalent circuit parameters. The feasibility of the optimized model was verified through finite element analysis (FEA). The study concluded that the optimized rotor geometry reduced core losses and generated an acceptable pull-out torque with lower stator current. It also had a significant impact on the motor’s synchronization capability. Additionally, in a FEM-based comparative study demonstrated that rotor bar design, including the number of rotor slots and bar material, significantly influences the torque profile and efficiency characteristics of induction motors. This highlights the importance of rotor geometry optimization for performance enhancement^9^. Bui Minh Dinh^10^ presented a design optimization study of an LSPMSM targeting maximum efficiency and starting torque capability. A Genetic Algorithm (GA) was employed to solve the defined optimization problem. The optimized design was manufactured and tested, confirming the validity of the design optimization approach. Hamidreza Behbahanifard and Alireza Sadoughi^11^ investigated the reduction of cogging torque. This phenomenon negatively impacts motor performance by increasing acoustic noise, vibration, torque ripple, and speed fluctuations. They employed a magnet-shifting technique to minimize cogging torque. Comparative testing involved manufacturing rotors both with and without magnet-shifting. The results showed that cogging torque was reduced by 71% in the rotor design with magnet shifting. The study concluded that the magnet-shifting technique is an effective approach for reducing cogging torque in LSPMSMs.

To enhance the overall performance of the LSPMSMs, several studies have been conducted in the literature. A maximum-efficiency design presented in^12^. Rotor shape optimization for high efficiency was introduced in^13,14^, and reducing permanent magnet (PM) usage while enhancing synchronization capability was studied in^15^. In addition, various LSPMSM design methodologies and optimization techniques were reviewed in^16^. GA are widely adopted to improve the performance of LSPMSMs^6,17,18^. Maximizing the efficiency, power factor, and starting torque of LSPMSMs is primarily considered the objective of optimization studies^19–21^.

A considerable number of studies in the literature have focused on the design, performance improvement, and optimization of LSPMSMs using various techniques. However, most of these studies have involved designing a new motor from scratch, which typically involves fewer design constraints and therefore offers a more flexible and less restrictive design process. In contrast, the present study addresses a more challenging problem: upgrading an existing IE1 efficiency class induction motor to the IE4 efficiency class solely by replacing the rotor, while keeping the stator, frame, and winding unchanged.

To the best of the authors’ knowledge, based on an extensive literature review, no previous study has been found that achieves an IE1 to IE4 efficiency upgrade through rotor replacement alone, which highlights the novelty of this work. It is also important to note that IE4 efficiency thresholds vary across different rated power and speed levels, which inherently affects other key performance metrics such as power factor and starting torque. Due to these variations, direct numerical comparisons with previously published LSPMSM designs operating at different power ratings and speeds are not straightforward.

Nonetheless, four critical benchmarks can be used to assess the effectiveness of an LSPMSM design in such retrofitting scenarios: (i) meeting the minimum efficiency requirement defined by the IE4 standard for the specified power and speed, (ii) achieving a starting torque level comparable to that of the original induction motor, (iii) maintaining stable synchronization under grid voltage fluctuations, and (iv) ensuring minimal use of permanent magnet material to reduce cost and resource dependency. All these criteria have been successfully met in the proposed design, thereby confirming the technical viability and performance robustness of the approach.

In this study, a design optimization process was conducted using the MOGA algorithm to develop an LSPMSM that meets the IE4 efficiency class. A conventional squirrel-cage induction motor with IE1 efficiency class was used as the reference design. The optimization process was carried out simultaneously for efficiency, power factor, demagnetization performance, and starting torque, ensuring balanced transient and steady-state performance. As a result of the optimization process, six candidate designs were obtained. These designs were subsequently analyzed and compared using FEA. The evaluation included various performance criteria such as demagnetization prediction at different operating temperatures, synchronization capability, and starting torque performance under nominal, low, and high voltage conditions. Based on the results of the comparative analysis, optimal design was developed as a prototype. Its performance was validated through tests such as efficiency measurement, temperature rise, and locked-rotor tests. In addition to design optimization and verification studies, three cost scenarios were evaluated based on the optimal design. The scenarios are as follows: (*i*) replacing the rotor of an existing IE1 induction motor with the optimized IE4 LSPMSM rotor, (*ii*) purchasing a new IE4 LSPMSM as a first-time investment, and (*iii*) replacing an already operating IE1 induction motor with a new IE4 LSPMSM. The payback period for each scenario was subsequently calculated.

### Main contributions

This study presents a novel approach to upgrading an IE1 efficiency class induction motor to IE4 efficiency by replacing only the rotor structure, while keeping the stator and mechanical components unchanged. The main contributions of this research are summarized as follows:

I. Instead of designing a new motor from scratch, the study demonstrates that replacing only the rotor with an optimized LSPMSM rotor can significantly improve efficiency.
II. The rotor slot geometry, magnet placement, magnet dimensions, and core length were optimized using MOGA, ensuring maximum efficiency with minimal cost increase.
III. Six optimized designs were analyzed for demagnetization, synchronization, and starting torque capability, leading to the selection of Design C as the optimal candidate.
IV. Detailed FEA simulations confirmed that Design C achieved 92.15% efficiency, which was later experimentally validated as 91.95%.
V. A prototype LSPMSM was manufactured and tested, proving that IE4 efficiency levels can be achieved with minimal modifications to an existing IE1 class induction motor.
VI. Three industrial implementation scenarios were analyzed, showing that replacing only the rotor can provide fast payback and significant energy savings.

### Main objectives and novelty of the paper

The main objective of this study is to provide a practical and cost-effective solution for enhancing the energy efficiency of industrial motors by retrofitting existing IE1 class induction motors with optimized LSPMSM rotors. The originality and novelty of this research are highlighted as follows:

I. Unlike most studies that focus on designing new LSPMSMs from scratch, this work introduces a retrofit strategy that maintains the stator and housing untouched while achieving IE4 performance through rotor redesign.
II. The approach addresses practical industrial needs by enabling the reuse of existing motor housings and components, thus lowering manufacturing and implementation costs.
III. The proposed design balances efficiency, starting torque, power factor, and demagnetization resistance using MOGA, which is rarely considered collectively in previous literature.
IV. The optimized design was physically prototyped and tested under various operating conditions, confirming the practical feasibility of the method.
V. A detailed cost-benefit analysis was conducted across multiple implementation scenarios, providing industry-relevant insights into investment strategies and return on investment.

The paper is structured to systematically present the design, optimization, validation, and feasibility analysis of the proposed LSPMSM. Section I introduces the motivation behind achieving higher efficiency in electric motors, the regulatory framework driving efficiency improvements, and the industrial need for cost-effective solutions. Section II provides the analytical design equations used in the optimization process, including key parameters such as magnet placement, rotor slot geometry, and core length. Section III details the optimization methodology, where MOGA was applied to maximize efficiency while minimizing costs. Section IV presents a comparative performance evaluation of six optimized designs, focusing on demagnetization resistance, synchronization performance, and starting torque capability to identify the most suitable candidate. Section V discusses the finite element analysis (FEA) validation, prototype manufacturing, and experimental test results, verifying the accuracy of the optimization process. Section VI explores the economic feasibility of implementing the LSPMSM by analyzing three different payback scenarios, including rotor replacement and full motor replacement strategies. Finally, Section VII concludes the study by summarizing the key findings, highlighting the practical implications of the results, and suggesting future research directions for further efficiency improvements and industrial applications.

## Design of the LSPMSM

**Fig. 1:**
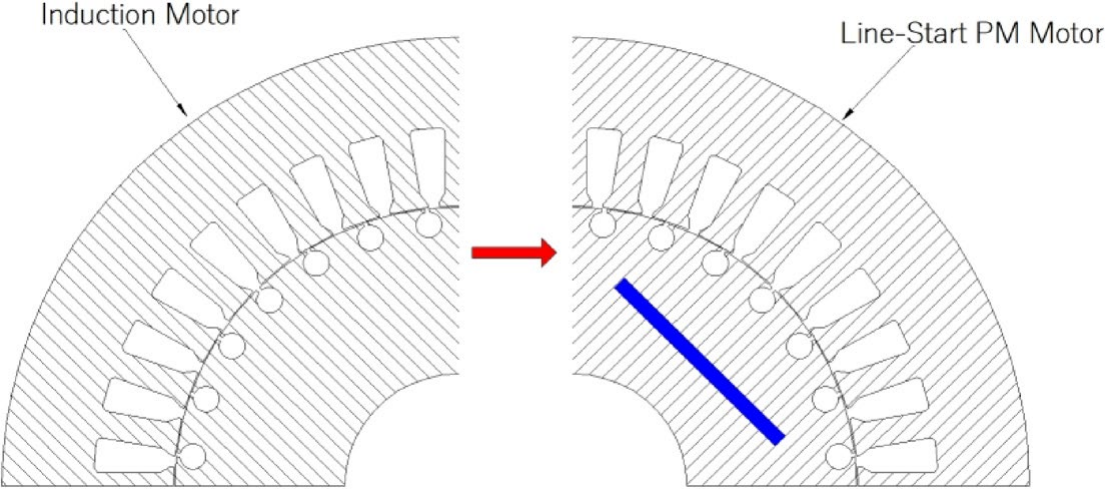
From IM to LSPMSM: Inserting PMs as the key difference.

The presence of both permanent magnets and short-circuited bars in the rotors of LSPMSMs adds complexity to the design process. Additionally, optimizing only the rotor of an induction motor, while keeping its stator inner and outer diameters and the number of stator slots fixed- to upgrade its efficiency class from IE1 to IE4 can be considered a highly challenging problem. Therefore, after obtaining the initial model using analytical calculation methods, optimization algorithms will be employed to achieve the desired performance criteria. The primary difference between induction motors and LSPMSMs arises from their rotor structure, as seen in Fig. 1. Unlike induction motors, the permanent magnets embedded in the rotor of LSPMSMs enable torque generation at synchronous speed. Meanwhile, the short-circuited bars in the rotor allow these motors to start under load. As a result, LSPMSMs exhibit characteristics of both induction motors and permanent magnet synchronous motors (PMSMs), making them a hybrid motor type.

In the analytical design process of electric motors, fundamental geometric parameters are first calculated. In the study, the equations used for sizing calculations will be presented; however, since only the rotor geometry is optimized based on a reference motor, these calculations will not be performed. The output coefficient expression for synchronous motors is given by the following equation^22^:

1 $$\:Output\:Coefft.\:\left({C}_{0}\right)=11\times\:{B}_{av}\times\:q\times\:{K}_{w}\times\:{K}_{c}\times\:{10}^{-3}$$

Here, $\:{B}_{av}$ represents the specific magnetic loading, $\:q$ denotes the specific electric loading, $\:{K}_{w}$ is the winding factor and, $\:{K}_{c}$ is the distribution factor. The specific electric and magnetic loading values are determined by the designer at the beginning of the design process. These values are calculated using the following equations:

2 $$\:Specific\:magnetic\:loading\:\left({B}_{av}\right)=\frac{P\times\:{\Phi\:}}{\pi\:\times\:D\times\:L}$$

3 $$\:Specific\:electric\:loading\:\left(q\right)=\frac{{I}_{a}\times\:{Z}_{a}}{\pi\:\times\:D}$$

Here, $\:P$ represents the number of poles, $\:{\Phi\:}$ denotes the magnetic flux per pole, $\:D$ is the stator inner diameter, $\:L$ is the rotor core length, $\:{I}_{a}$ is the armature current, and $\:{Z}_{a}$ is the number of armature conductors. A higher specific electric and magnetic loading leads to a more compact and cost-effective motor. However, increased values also result in higher heat generation, efficiency variations, and increased copper losses. These values are determined by the designer at the beginning of the design process, based on experience accumulated over many years^23^.

The volume of the motor, calculated based on the given equations for the output coefficient, is expressed in terms of the stator inner diameter and core length as follows:

4 $$\:{D}^{2}L=\frac{Kw}{{C}_{0}\times\:{n}_{s}}$$

Here, $\:Kw$ represents the motor output power, and $\:{n}_{s}$ denotes the synchronous speed. Once $\:{D}^{2}L$ is calculated to determine the active volume of the motor, the stator inner diameter and core length are separated to obtain the fundamental dimensions of the motor. After defining the stator volume, the next critical parameter to be determined is the stator slot geometry. The thickness of the stator teeth plays a crucial role in the distribution of magnetic flux and determines whether the core reaches saturation^24^. The stator tooth thickness can be calculated as follows, by considering the total magnetic flux passing through the stator teeth:

5 $$\:{S}_{tw}=\frac{{B}_{av}\times\:L}{{B}_{t}\times\:{L}_{i}}\times\:{\tau\:}_{s}$$

Here, $\:{B}_{t}$ represents the maximum flux density in the stator teeth, $\:{\tau\:}_{s}$ denotes the slot pitch, and $\:{L}_{i}$ is the effective stator core length. Additionally, based on the magnetic flux per pole, the stator yoke thickness $\:{S}_{bi}$ can be expressed as follows:

6 $$\:{S}_{bi}=\frac{{\Phi\:}}{{2\times\:B}_{ys}\times\:{L}_{i}}$$

$\:{B}_{ys}$ represents the maximum magnetic flux density in the stator. The air gap length, defined as the distance between the stator inner diameter and the rotor outer diameter, is a critical parameter affecting motor performance and can be calculated using the following equation:

7 $$\:{l}_{g}=4\times\:{10}^{-7}\frac{\pi\:\times\:D\times\:q\times\:{\tau\:}_{s}}{\left({\tau\:}_{s}-\frac{{w}_{s}/g}{5+{w}_{s}/g}\times\:{w}_{s}\right)\left(0.6-\left(0.8{B}_{r}P\right)\right)}$$

Here, $\:{B}_{r}$ represents the residual flux density in the permanent magnets, and $\:g$ denotes the physical air gap length. Based on the motor’s output power, the selected magnet material, the number of poles, and the operating frequency, the magnet volume can be calculated using the following equation:

8 $$\:{V}_{pm}={C}_{V}\frac{{P}_{out}}{Pf{B}_{r}{H}_{c}}$$

In the given equation, $\:{C}_{V}$ is a constant that can be selected within the range of 0.54 to 3.1. $\:{H}_{c}$ represents the coercive force, and $\:f$ denotes the motor frequency. Once the magnet volume is determined, the magnet length is selected based on the rotor’s inner and outer diameters^25^.

9 $$\:\frac{{R}_{id}}{2}\le\:mw\le\:\frac{{R}_{od}}{2}$$

$\:{R}_{id}$ represents the rotor inner diameter, and $\:{R}_{od}$ denotes the rotor outer diameter. The magnet thickness is determined by considering armature reaction and magnetic saturation and is calculated as follows:

10 $$\:{m}_{t}=\frac{{g}_{m}{k}_{f}{k}_{r}{\mu\:}_{rm}}{{B}_{r}{k}_{l}{k}_{f}-{B}_{g}}$$

**Fig. 2:**
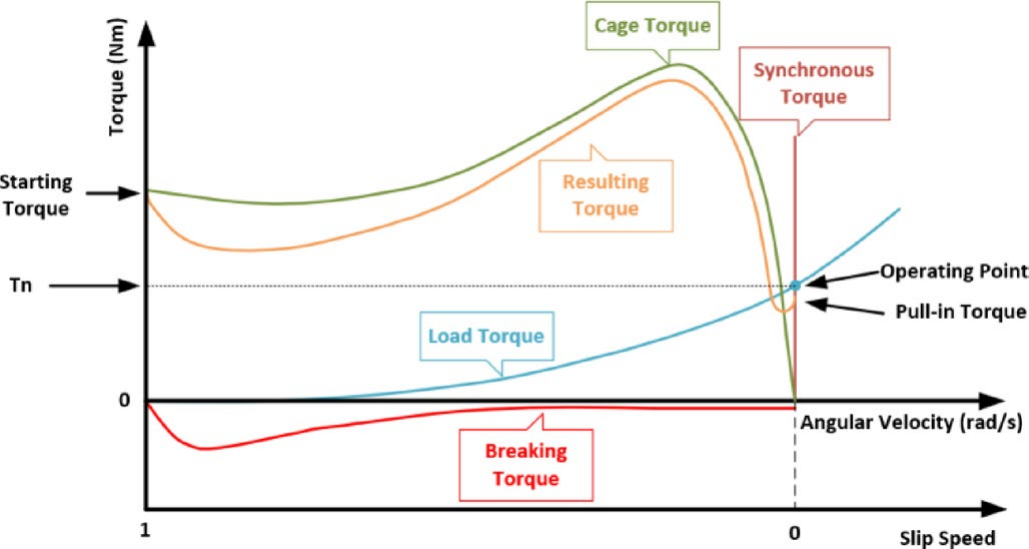
Torque components of the LSPMSM^7^.

Here, $\:{g}_{m}$ represents the maximum air gap length, $\:{k}_{f}$ is the ratio of the permanent magnet cross-section to the air-gap cross-section, $\:{k}_{r}$ is the reluctance coefficient, $\:{\mu\:}_{rm}$ is the relative permeability, and $\:{k}_{l}$ is the leakage coefficient. The permanent magnets used in the rotor of LSPMSMs influence both the transient and steady-state performance of the motor. Figure 2 illustrates the torque contributions of the magnets and short-circuited bars during the transition from startup to synchronous speed in LSPMSMs, along with the resultant torque.

In LSPMSMs, the rotor short-circuited bars generate torque until the motor reaches synchronous speed. Once the motor reaches synchronous speed, the rotor short-circuited bars no longer contribute to torque generation, similar to induction motors. However, the rotor slot geometry directly affects PM magnetization. When the rotor slot cross-section is reduced, eddy currents decrease due to increased rotor resistance and magnetic flux density. Therefore, optimizing the rotor geometry is crucial for enhancing the performance of LSPMSMs^6^. In LSPMSMs, not only the short-circuited bars but also the magnet geometry and the *BH*_*max*_ product of the permanent magnets are critical factors for motor performance. If the PM magnetization is either insufficient or excessive, synchronization failure may occur. If the magnetization is too low, the motor will be unable to generate the required torque at synchronous speed, preventing synchronization. Conversely, if the magnetization is too high, the braking torque may exceed the cage torque, preventing the motor from starting or achieving synchronization. The braking torque in LSPMSMs can be expressed using the following equation^25^:

11 $$\:{T}_{b}=\frac{3p{R}_{s}{(1-s)}^{2}{E}_{0}^{2}}{2{\omega\:}_{s}(1-s)}.\frac{{R}_{s}^{2}+{X}_{qs}^{2}{(1-s)}^{2}}{{{(R}_{s}^{2}+{X}_{qs}{X}_{ds}{(1-s)}^{2})}^{2}}$$

Here, $\:s$ represents the slip, $\:{R}_{s}$ denotes the stator resistance,$\:{E}_{0}$ is the induced voltage at synchronous speed, $\:{\omega\:}_{s}$ is the synchronous electrical speed, $\:{X}_{qs}$ is the stator reactance in the q-axis, and $\:{X}_{ds}\:$is the stator reactance in the d-axis. However, since the cage torque disappears once the motor reaches synchronous speed, the electromagnetic torque expression can be defined as follows^26^:

12 $$\:{T}_{em}=\frac{3{U}_{eM}U}{{\varOmega\:X}_{d}}sin\theta\:-\frac{3{U}^{2}}{2\varOmega\:}\left(\frac{1}{{X}_{d}}-\frac{1}{{X}_{q}}\right)sin2\theta\:$$

In LSPMSMs, the starting torque is generated by the rotor bars. However, once the motor reaches synchronous speed, meaning in the steady-state operating condition, the rotor bars no longer contribute to torque generation. Instead, in the steady-state condition, the rotor bars act as flux barriers, influencing the demagnetization performance of the permanent magnets. The rotor slot parameters are calculated using the following Eq^6^. :

13 $$\:{H}_{r2}={A}_{b}-\frac{\pi\:\left({B}_{r1}^{2}+{B}_{r2}^{2}\right)}{2\left({B}_{r1}^{2}+{B}_{r2}^{2}\right)}$$

Here, $\:{A}_{b}$ represents the rotor slot cross-section.

## Design optimization of the LSPMSM

In this section, the technical specifications of the reference motor, design objectives, optimization process, the upper and lower limits of design parameters, and the objective function are presented in detail.

### Reference IM and proposed LSPMSM specifications

The reference motor used in this study is a standard induction motor classified as IE1 efficiency class according to the IEC 60034-30-1 standard. The target specifications of the LSPMSM to be designed in this study, along with the characteristics of the reference motor, are presented in Table 2.

**Table 2:** Main specifications of the reference IM and LSPMSM.

	Reference IM	LSPMSM
Output Power (kW)	5.5	5.5
Number of Poles	4	4
Efficiency (%)	84.1	> 91.9
Efficiency Class	IE1	IE4
Power Factor	0.844	>=0.95
Current (A)	11.77	< 9.5
Duty	S1	S1
Change in Temperature Rise (PT100 ∆T)	84	< 50

For the designed LSPMSM, the rated power, number of poles, operating duty, stator outer diameter, stator inner diameter, and stator slot geometry were kept the same as those of the reference motor. The geometric parameters of the reference IE1 efficiency class induction motor and the material properties of the permanent magnet selected for the LSPMSM rotor are presented in Table 3.

**Table 3:** Reference IM and LSPMSM parameters.

Parameter	Reference IM	LSPMSM
Stator Outer Diameter (mm)	200	
Stator Inner Diameter (mm)	125	
Rotor Outer Diameter (mm)	124.2	
Stack Length (mm)	125	Variable
Stator Number of Slot	36	
Rotor Number of Slot	28	
Stator Winding Type	Lap	
Winding Layer	2	
Conductor per slot	44	
Coil Pitch	9	
Core Material	M400-50 A	
Magnet Material	N/A	N40-UH

As shown in Table 3, the fundamental geometric dimensions of the reference motor and the LSPMSM, such as -stator outer diameter, stator inner diameter, and rotor inner diameter- remain the same. Among the key parameters that affect motor volume, only the core length was considered a variable during the design process. As a result, the design optimization study aims to achieve a Super Premium efficiency (IE4) LSPMSM by modifying only the rotor structure of a 5.5 kW standard efficiency (IE1) induction motor. With the efficiency level increasing from IE1 to IE4, the motor’s temperature rise is expected to be lower than that of the reference motor. In this case, using a lower-loss fan could enable the motor to operate with higher efficiency without thermal overloading. In the study, windage and friction losses were assumed to be constant.

### Optimization study

The design of electrical machines is based on electromagnetic theory, making electromagnetic design critically important^27^. The electromagnetic design process requires solving a multi-objective optimization problem, in which torque and efficiency maximization must be achieved simultaneously with cost minimization. To address such challenges in the electromagnetic design process, optimization algorithms serve as a powerful tool. In Section II, the design equations for the LSPMSM were provided. Based on these equations, a design optimization study was conducted for the following key parameters: rotor slot geometry, which influences transient performance; magnet geometry, which affects steady-state performance; core length, which impacts the output coefficient and thus the thermal load; and number of winding turns, which influences all performance parameters of the motor. The multi-objective optimization study was carried out considering these parameters. The performance criteria and their weights in the objective function are expressed by Eq. (14), and the cost of the optimization is calculated by Eq. (15).

14 $$\:f\left(x\right)=\left\{\begin{array}{c}{\:f}_{1}\left(x\right)=Efficiency\:\ge\:\:92.3,\:\:\:\:weight=30\\\:{f}_{2}\left(x\right)=\:Magnet\:Weight\:\le\:\:0.6,\:\:\:\:weight=30\\\:{f}_{3}\left(x\right)=Power\:Factor\ge\:\:0.95,\:\:\:\:\:weight=10\\\:{\:f}_{4}\left(x\right)=Starting\:Torque\:\ge\:\:100,\:\:\:\:\:weight=10\\\:{\:f}_{5}\left(x\right)=Current\:Density\le\:\:5.5,\:\:\:\:\:\:weight=10\\\:{\:f}_{6}\left(x\right)=Rotor\:Teeth\:Flux\:Density\le\:\:1.25,\:\:\:\:weight=5\\\:{\:f}_{7}\left(x\right)=Stator\:Teeth\:Flux\:Density\le\:\:1.25,\:\:\:\:weight=5\end{array}\right.$$

15 $$\:Cost=\sum\:_{i=1}^{7}({f}_{i}.{w}_{i})$$

Here, when determining the efficiency value, additional losses and potential simulation error margins were considered. Although the IEC 60034-2-1 standard specifies a minimum efficiency of 91.9% for a 50 Hz, 4-pole, 5.5 kW motor in the IE4 efficiency class, the efficiency threshold in the optimization study was set to 92.3% to account for these factors. Since the primary objective of this study is to upgrade an IE1 efficiency class motor to the IE4 efficiency class, the weight of motor efficiency in the objective function was set to 30%. From a cost-independent perspective, achieving IE4 efficiency is a relatively straightforward problem. However, designing a high-efficiency and cost-effective electric motor with permanent magnets in the rotor is a more complex challenge. Therefore, the magnet quantity, as the most expensive active material used in the motor, was also assigned a 30% weight in the objective function. Additionally, power factor, starting torque, and stator current density were included in the optimization as key performance criteria for both asynchronous and synchronous operation, with each parameter assigned a 10% weight. Finally, rotor and stator flux densities were considered to prevent core saturation, with each assigned a 5% weight in the objective function.

**Fig. 3:**
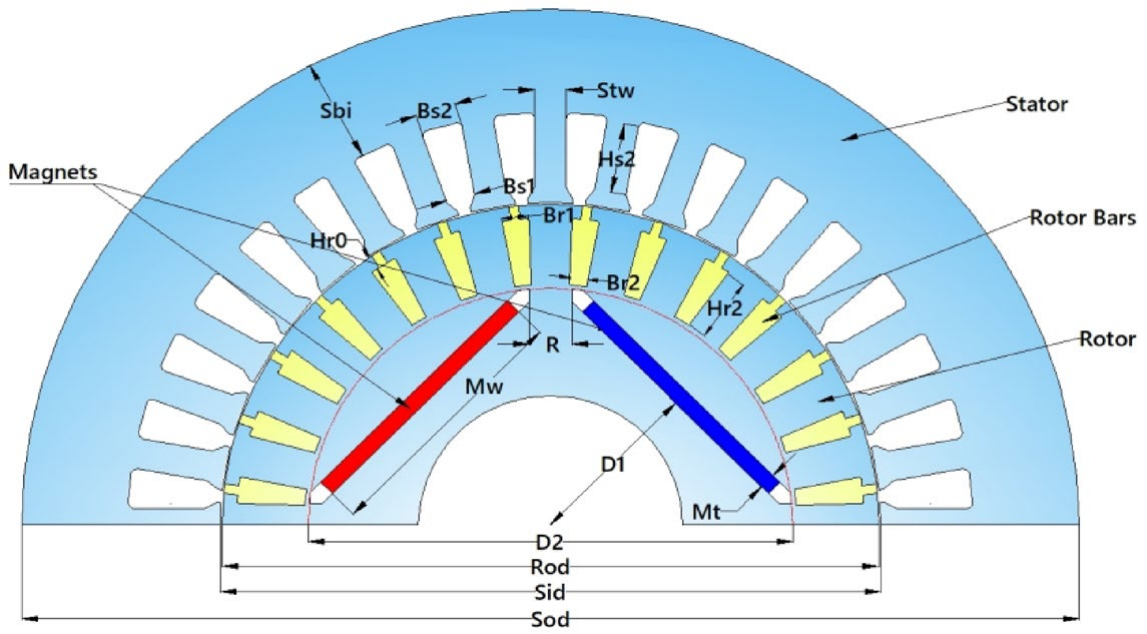
Geometrical representation of design variables.

During the design optimization process, the Multi-Objective Genetic Algorithm (MOGA) was employed to achieve a design that meets the performance parameters specified in Eq. (12). MOGA, integrated within the Ansys Electronics software package for optimizing electric machines, is a hybrid GA-based global optimization method derived from the Non-Dominated Sorting Genetic Algorithm-II (NSGA-II). It is specifically designed for solving multi-objective function problems, ensuring an optimal trade-off between competing design criteria^28,29^. The geometric design parameters of the motor optimized using the MOGA algorithm are presented in Fig. 3.

The upper and lower limits of the design variables, which influence both steady-state and transient performance, were selected within physically feasible ranges. These limits are presented in Table 4.

**Table 4:** Upper and lower limits of the design variables.

Variable	Description	Initial	Min	Max
Rated Voltage	Motor rated voltage	400	200	500
Mw	PM width	51	30	51.5
Mt	PM thickness	4	2.5	5
D2	Diameter of the flux barriers	91	89.5	92
Hr0	Height of the rotor slot opening	2.8	2	7
Hr2	Cage slot tooth height	12.11	12.91	7.91
Br1	Cage slot tooth upper width	7.6	4	9
Br2	Cage slot tooth lower width	5.7	2.1	7.1
L	Stack length	140	100	150
Sod	Stator Outer Diameter	200	Same as reference IM	
Sid	Stator Inner Diameter	125		
Rod	Rotor Outer Diameter	124.2		
Stw	Stator tooth width	6.1		
Hs2	Stator slot tooth height	13.22		
Sbi	Stator back iron	20.05		

**Fig. 4:**
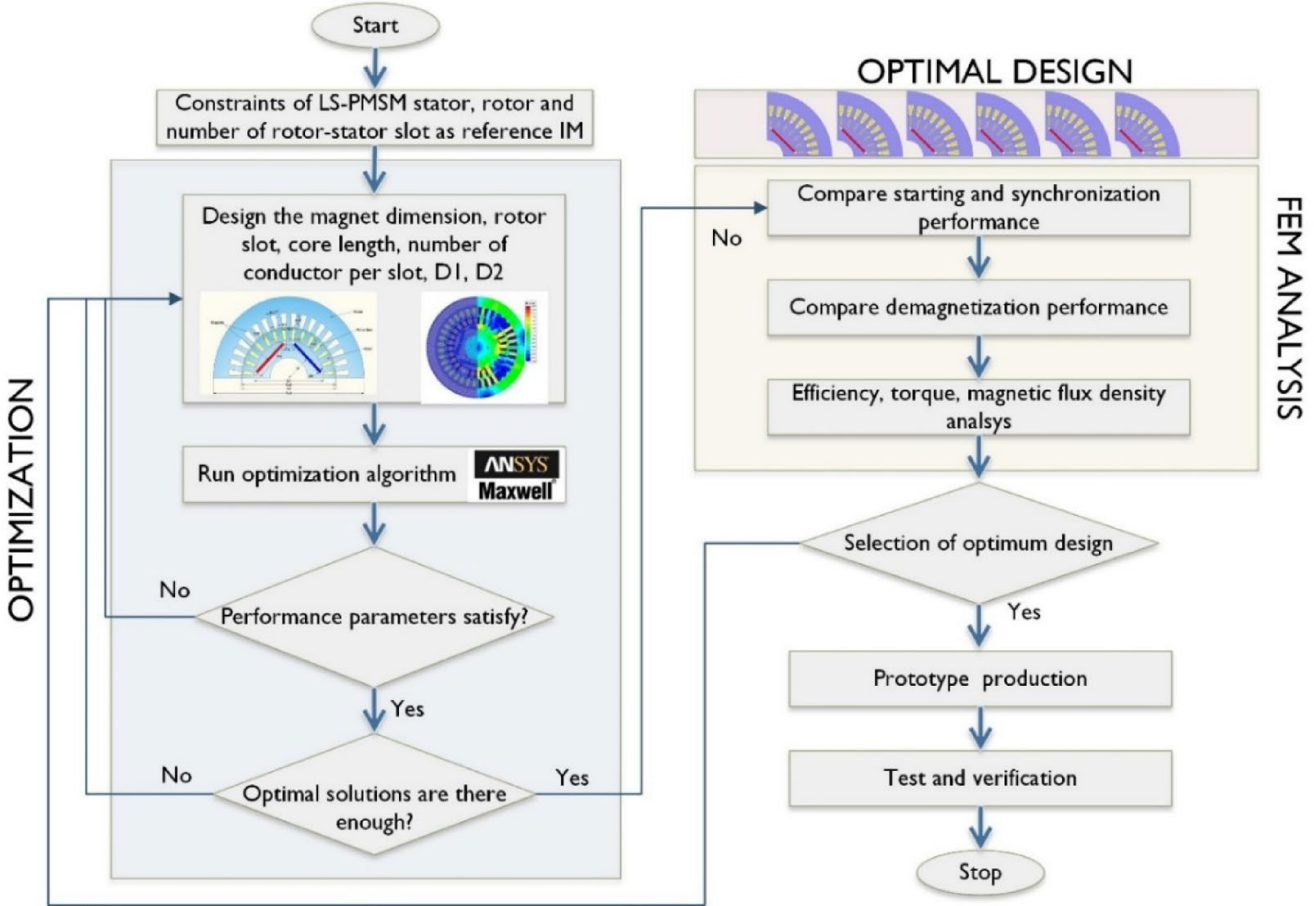
Flowchart of the design optimization.

In the study, the stator inner and outer diameters, rotor outer diameter, and stator slot geometry were kept unchanged by using the reference motor’s dimensions. The magnet geometry, magnet position within the rotor, and geometric components of the short-circuited bars were selected as optimization parameters. During the design optimization process, ANSYS Maxwell software, which is widely used for electric machine design, was utilized. The optimization study was conducted using the MOGA algorithm within the ANSYS RMxprt package. The entire design process is illustrated in the flowchart presented in Fig. 4.

The design process begins with the determination of fundamental motor parameters. Using these parameters, an initial motor model is obtained through analytical calculation methods. The initial motor model is then optimized using the MOGA algorithm, ensuring that the design parameters listed in Table 4 achieve the performance values specified in Eq. (12). To select the most suitable design candidates from the optimized models, FEA simulations were conducted to evaluate: (a) demagnetization characteristics, (b) synchronization performance, and (c) starting torque capability. After conducting comparative analyses, an optimal LSPMSM design was identified. The motor was then manufactured, and design validation tests were performed.

## Comparative analysis of optimization results

In this section, the results of the optimization process are presented along with the comparative evaluation of key performance aspects. The analysis includes demagnetization characteristics, synchronization performance, and starting torque capability. The findings from these analyses are compared to assess the effectiveness of the optimized LSPMSM design, ensuring that it meets the IE4 efficiency class while maintaining reliable operational performance.

### Optimization results

**Fig. 5:**
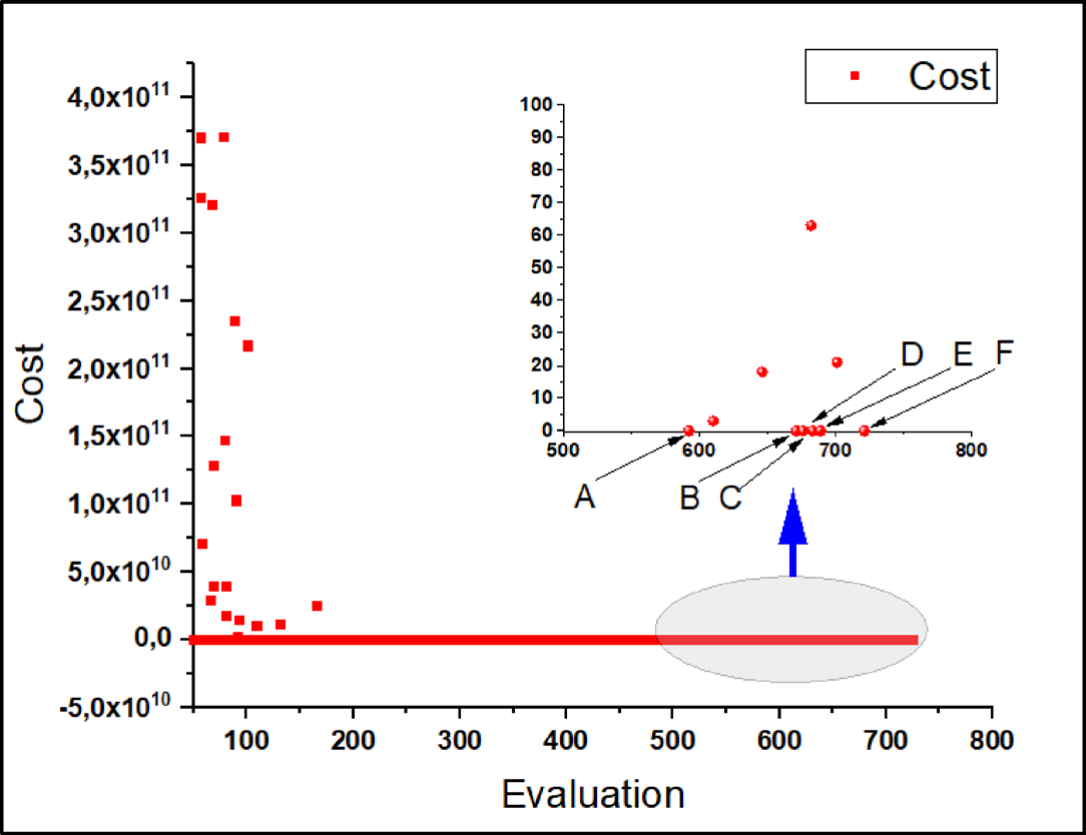
Six optimal solutions obtained by MOGA.

During the design optimization process, both the Genetic Algorithm (GA) (random search) and the Multi-Objective Genetic Algorithm (MOGA) were applied separately. In both approaches, the same objective function and design parameters were utilized. After approximately 5000 iterations, only two solutions met the required performance criteria using GA. On the other hand, after 720 iterations, six solutions satisfying the desired performance parameters were obtained using MOGA, as shown in Fig. 5. These results indicate that MOGA provided a significantly more efficient optimization process, achieving a higher number of valid solutions with fewer iterations compared to the standard GA. It is concluded that MOGA achieved the optimal solution more efficiently compared to GA.

**Fig. 6:**
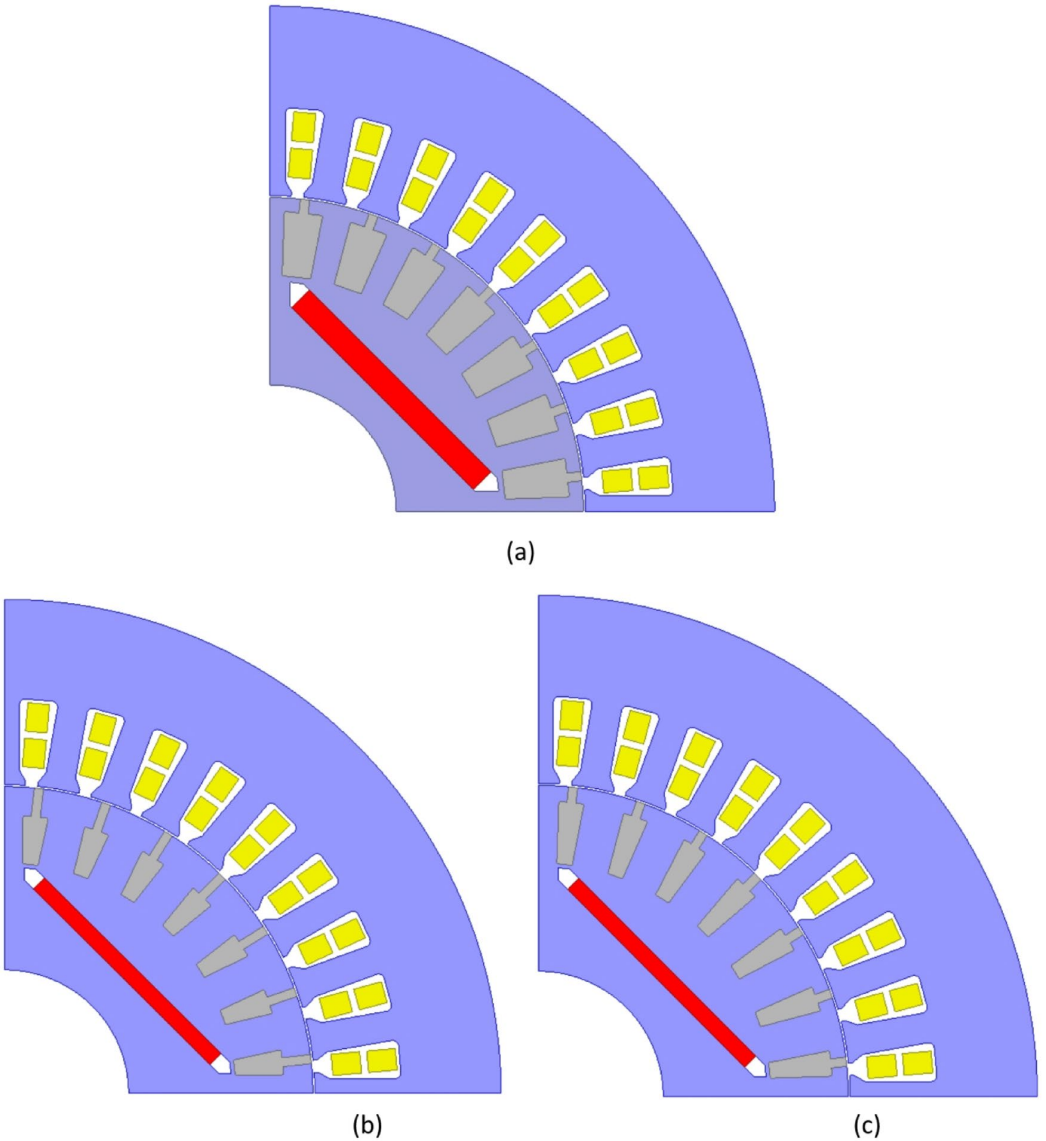

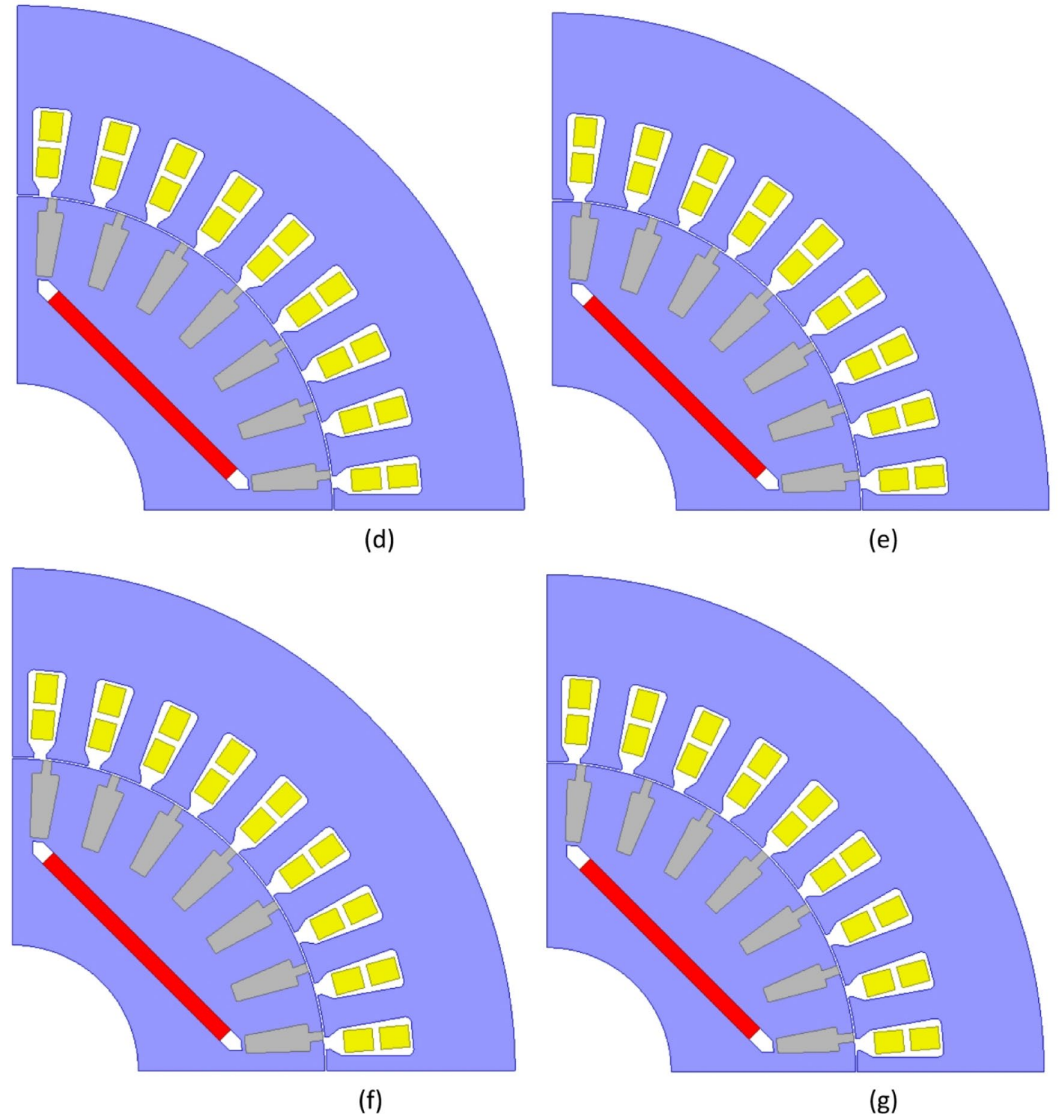
Rotor and stator geometry (**a**) Initial design, (**b**) Design A, (**c**) Design B, (**d**) Design C, (**e**) Design D, (**f**) Design E, (**g**) Design F.

The optimization was performed on a computer equipped with an Intel i9-9900K processor (16 MB cache, 2.1 GHz) and 64 GB RAM. In 12 min, MOGA completed 720 iterations, yielding six optimal solutions that met the required performance criteria. This efficiency is attributed to MOGA’s ranking mechanism, in which feasible solutions are consistently prioritized over non-feasible ones, leading to faster convergence to optimal results^27^. The six optimal designs *(A*,* B*,* C*,* D*,* E*,* F)* obtained using MOGA and the initial design are visually represented in Fig. 6, showing their corresponding one-quarter geometries.

Upon examining the geometric parameters of the six candidate designs, it was observed that magnet thickness, core length, voltage per turn, and magnet position within the rotor exhibited minimal variation among the designs. The most notable difference among the six models was found in the rotor slot geometry. The design parameters of the initial and the six optimized designs are presented numerically in Table 5.

**Table 5:** Parameters of the six optimal designs.

	Initial Design	Design A	Design B	Design C	Design D	Design E	Design F
Rated Voltage [V]	400	373.00	368.36	372.79	372.91	373.16	367.42
Core Length [mm]	140	126.98	123.09	126.98	124.26	126.99	126.49
Magnet Width [mm]	51	50.14	50.14	49.88	49.10	49.88	48.14
Magnet Thickness [mm]	5	3.09	3.09	3.12	3.12	3.10	3.10
D2	91	91.61	91.61	91.66	91.66	91.67	91.61
O1	31.50	33.41	33.40	33.37	33.37	33.39	33.39
Hr0	2.80	5.77	3.10	2.67	2.86	2.67	3.16
Hs01	0	0	0	0	0	0	0
Hr1	0.40	0.40	0.40	0.40	0.40	0.40	0.40
Hr2	12.11	9.14	11.80	12.24	12.04	12.24	11.75
Br0	1.83	1.83	1.83	1.83	1.83	1.83	1.83
Br1	7.60	5.04	5.04	5.10	5.47	5.47	4.95
Br2	5.70	3.14	3.14	3.20	3.57	3.57	3.05
Rs	0.20	0.20	0.20	0.20	0.20	0.20	0.20

**Table 6:** Comparison of the optimal designs.

	Design A	Design B	Design C	Design D	Design E	Design F
Efficiency (%)	92.31	92.30	92.35	92.36	92.35	92.31
Magnet Weight (kg)	0.583	0.566	0.585	0.573	0.581	0.558
Power Factor	0.951	0.950	0.955	0.953	0.957	0.950
Starting Torque (Nm)	116.0	127.1	129.8	125.6	126.7	124.9
Current Density (A)	4.719	4.781	4.697	4.707	4.686	4.796
Rotor Teeth Flux Density (T)	1.163	1.113	1.11	1.167	1.180	1.056
Stator Teeth Flux Density (T)	1.121	1.115	1.11	1.107	1.123	1.069

Each design parameter has a direct impact on motor performance. By evaluating Tables 5 and 6 together, the following key observations and conclusions have been drawn:

- Rotor and Stator Tooth Magnetic Flux Densities: **All candidate designs** exhibit very similar values with minimal differences.
- Current Density: **Design C and D** have the lowest current density values, indicating that these designs are thermally more favorable compared to the others.
- Power Factor: **Design C and E** provide the highest power factor with a slight difference, making them strong candidates for optimal design.
- Efficiency: **Design C**,** D**,** and E** achieve the highest efficiency values with minimal variation.
- Rotor Slot Depth: **Design C** features the deepest rotor slots, whereas **Design A** has the shallowest. Since slot depth is directly related to the motor’s starting torque capability, this suggests a significant correlation between slot geometry and startup performance.
- Braking Torque and Magnet Usage: Despite **Design C** having the highest amount of magnet material, the braking torque was successfully managed. The rotor slot geometry significantly influenced starting performance, demonstrating its critical role in LSPMSM design.

Upon evaluating the analytical analysis results for the six candidate designs, it is evident that Designs C, D, and E exhibit superior performance compared to Designs A, B, and F. However, selecting the optimal design cannot be based solely on analytical analysis results. To determine the best design, further evaluations must be conducted on demagnetization performance, synchronization capability, and starting torque performance. These performance analyses are presented in detail in the next section.

### Comparison of demagnetization performances

In permanent magnet (PM) motors, demagnetization of the magnets can significantly impact motor performance^28^. Therefore, evaluating the demagnetization characteristics is a crucial step in assessing motor performance.

**Fig. 7:**
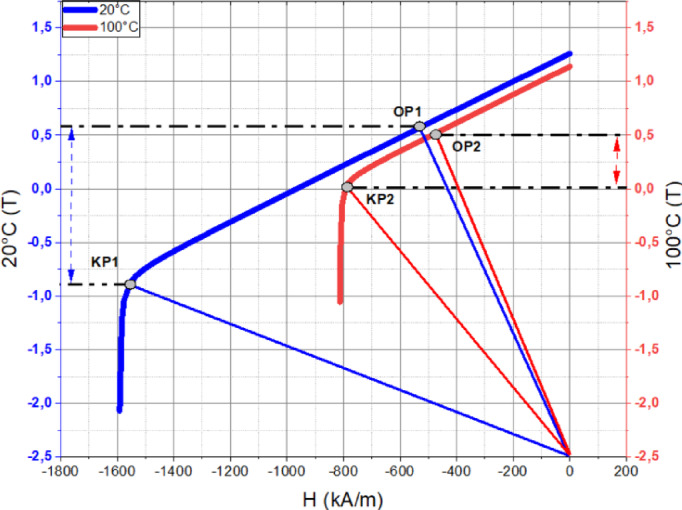
B-H curve of the N40-UH magnet.

**Fig. 8:**
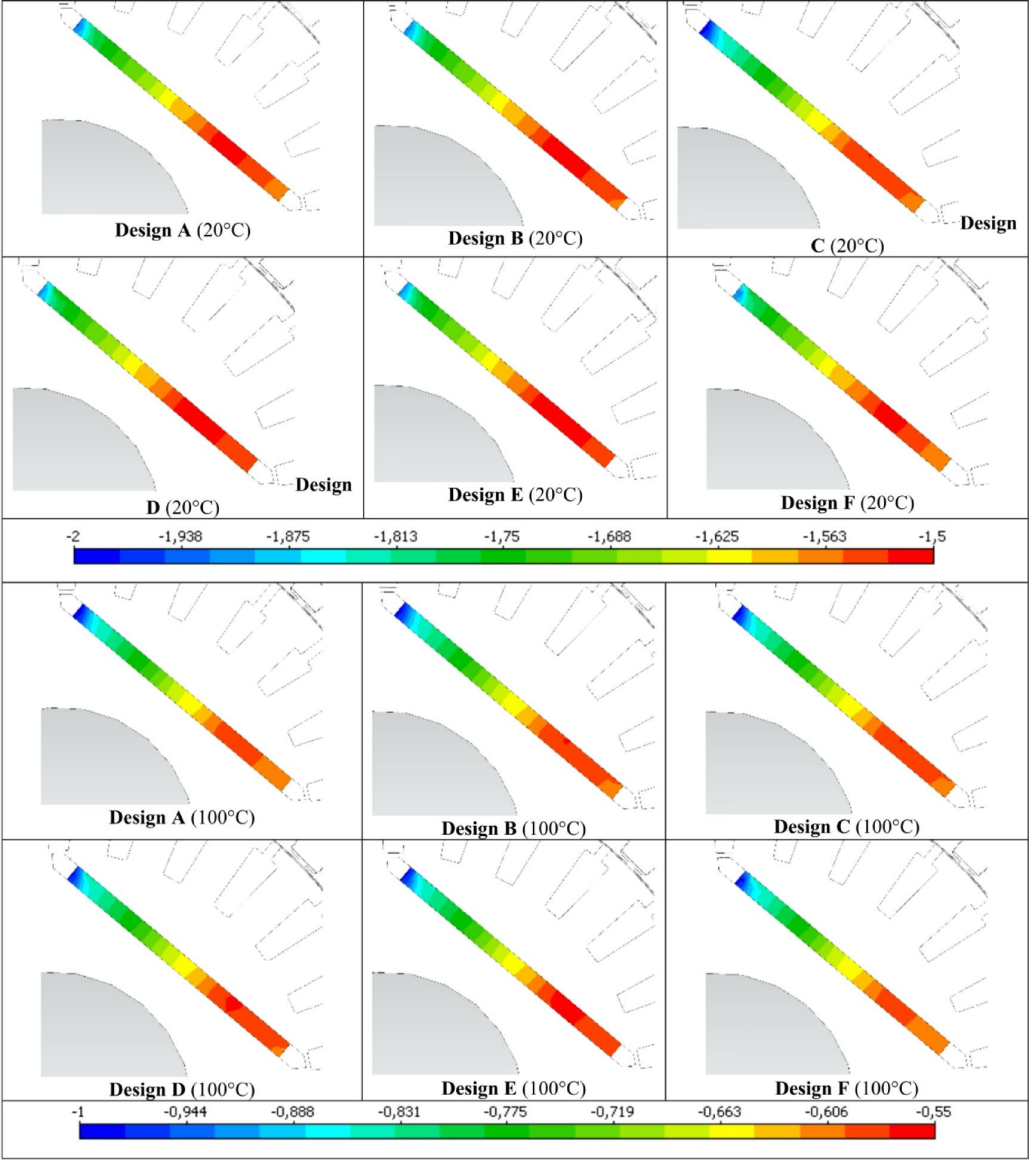
Comparison of steady-state demagnetization prediction results.

To determine the most suitable design, demagnetization prediction analyses were conducted for the six candidate motors using MotorSolve software. Since magnet temperature influences the operating point of the magnet, demagnetization prediction analyses were performed separately at 20 °C and 100 °C. The B-H curves of the N40-UH magnet at 20 °C and 100 °C are presented in Fig. 7. In the B-H curves provided for the N40-UH magnet at 20 °C and 100 °C, KP1(y) and KP2(y) represent the knee points of the B-H curve, indicating the demagnetization threshold beyond which the magnet’s performance significantly deteriorates. Op1(y) and Op2(y) represent the operating points, showing the actual working conditions of the magnet at the respective temperatures. These values are critical for assessing the risk of demagnetization under different operating temperatures and ensuring the reliability of the optimized motor designs. MotorSolve software predicts demagnetization based on the difference between the knee point (Kp) and the operating point (Op) of the B-H curve. At 20 °C, the Kp1(y) – Op1(y) value is approximately − 1.5 T, at 100 °C, the Kp2(y) – Op2(y) value is around − 0.5 T, as shown in Fig. 7. A negative Kp(y) – Op(y) value indicates that the magnet is not demagnetized. The smaller this difference, the lower the risk of demagnetization. However, if the scale value is positive (Kp(y) < Op(y)), it means that the operating point has fallen below the knee point, indicating that the magnet has undergone demagnetization. The demagnetization prediction analysis results obtained using this method are presented comparatively for 20 °C and 100 °C in Fig. 8.

The demagnetization prediction analyses for the six candidate designs were conducted at rated speed, under 150% load, and at both 20 °C and 100 °C. When examining the results at 20 °C and 100 °C separately, it was observed that the demagnetization characteristics of all candidate designs are quite similar. Although demagnetization performance alone is not the sole determining factor in selecting the optimal design, it is confirmed that all six candidate designs are suitable in terms of demagnetization performance.

### Comparison of synchronization performances

One of the most critical features of LSPMSMs is their ability to start under load conditions, enabled by the short-circuited bars in the rotor. To evaluate this capability, the six optimized designs were analyzed for load-starting performance at rated voltage using ANSYS Maxwell. The results were then compared with those of a standard induction motor of the same power rating, featuring both aluminum and copper rotor designs.

**Fig. 9:**
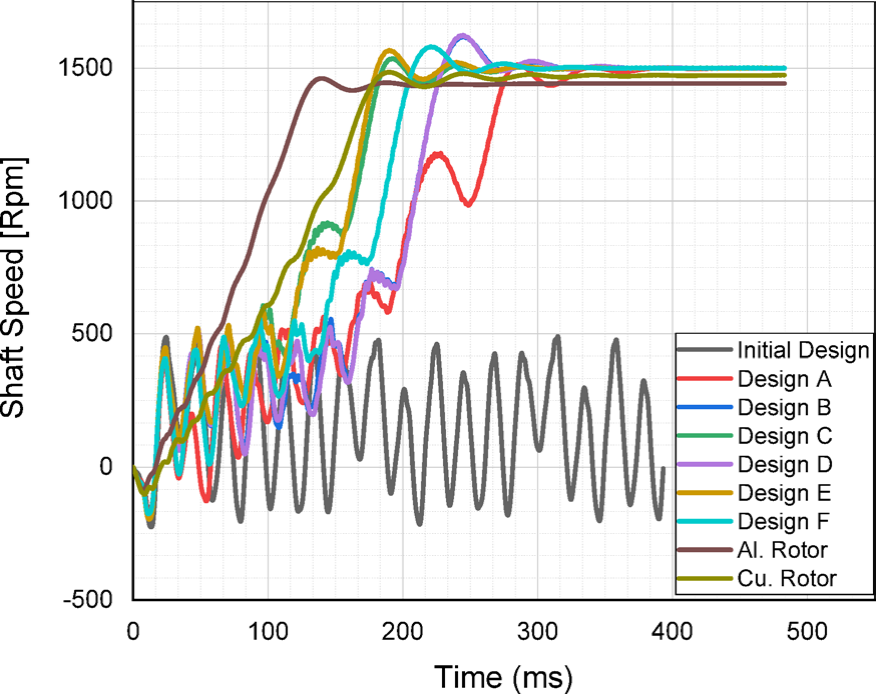
Comparison of the start-up and acceleration profiles.

The synchronization performance of the candidate designs was analyzed under their rated load of 35 Nm. As clearly illustrated in Fig. 9, while the initial design fails to synchronize under rated load conditions, all candidate designs obtained through the optimization process successfully achieved synchronization at rated load. Additionally, a transient analysis was conducted for the reference induction motor, considering both aluminum and copper rotor conductors. These results are presented in Fig. 9. When comparing the acceleration times from startup to rated speed, the induction motor with an aluminum rotor reached synchronization the fastest.

**Fig. 10:**
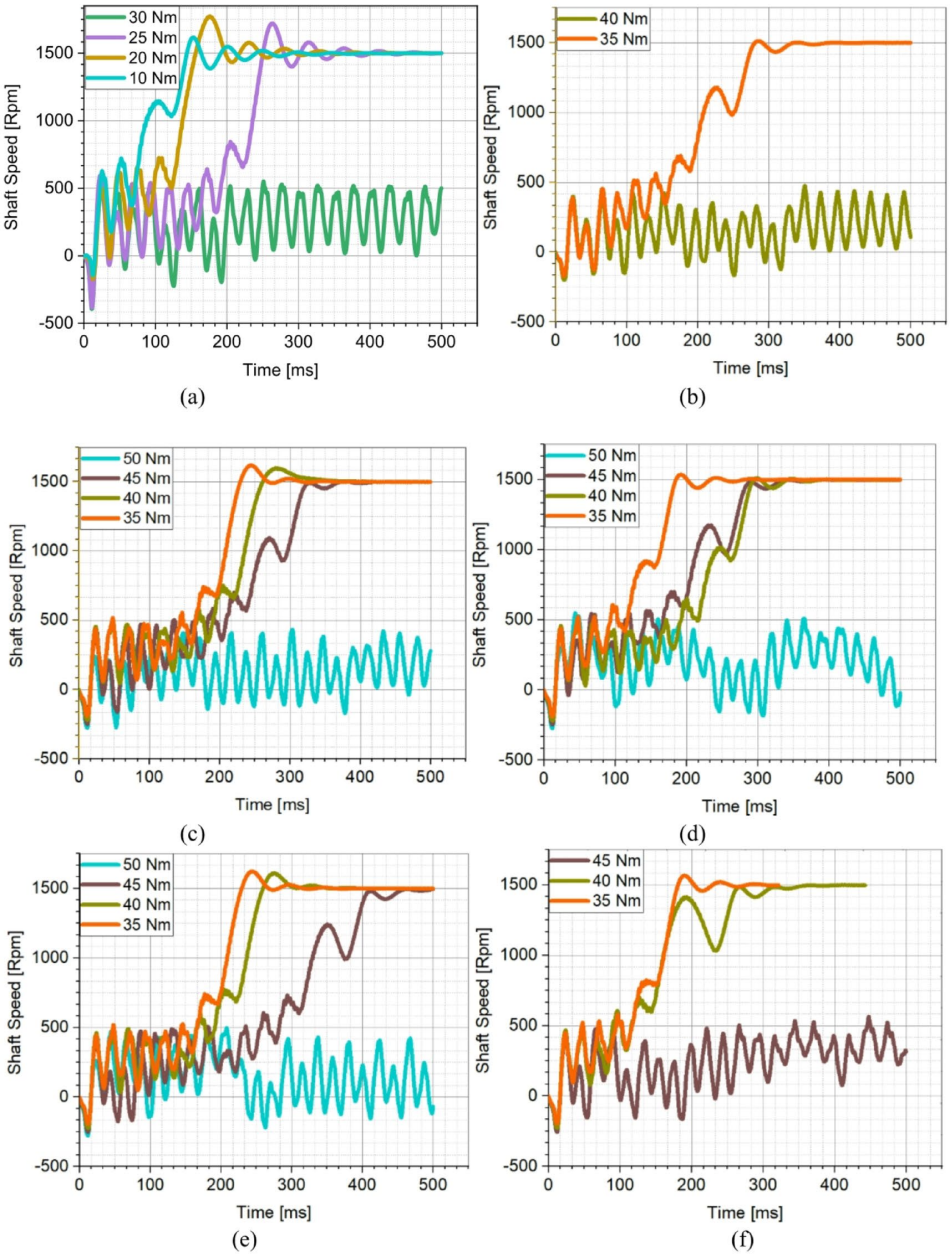

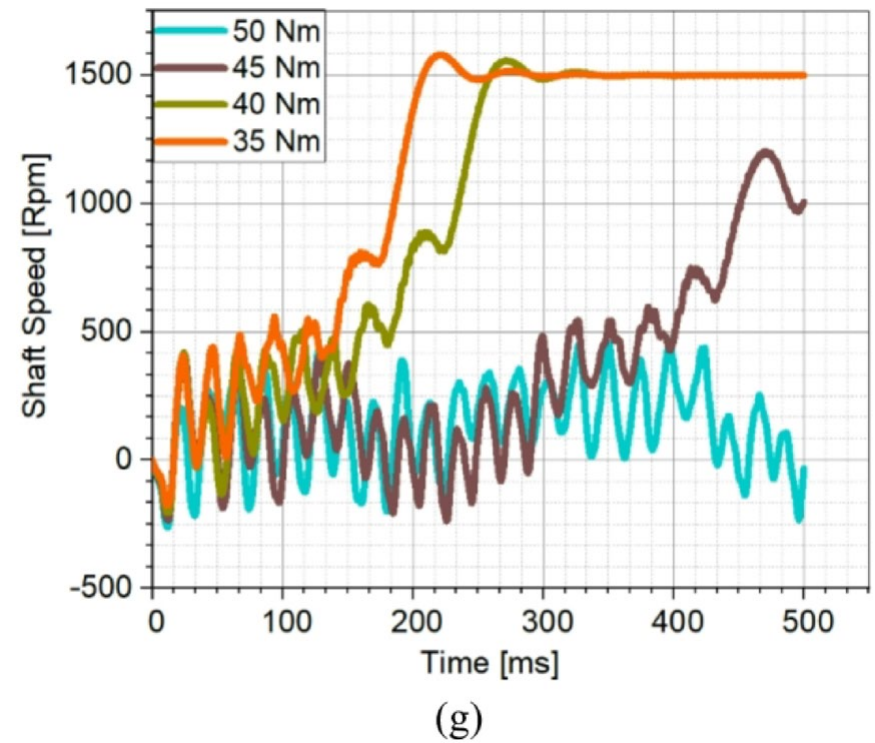
Evaluation of synchronization performance under different loads (**a**) Initial Design, (**b**) Design A, (**c**) Design B, (**d**) Design C, (**e**) Design D, (**f**) Design E, (**g**) Design F.

**Fig. 11:**
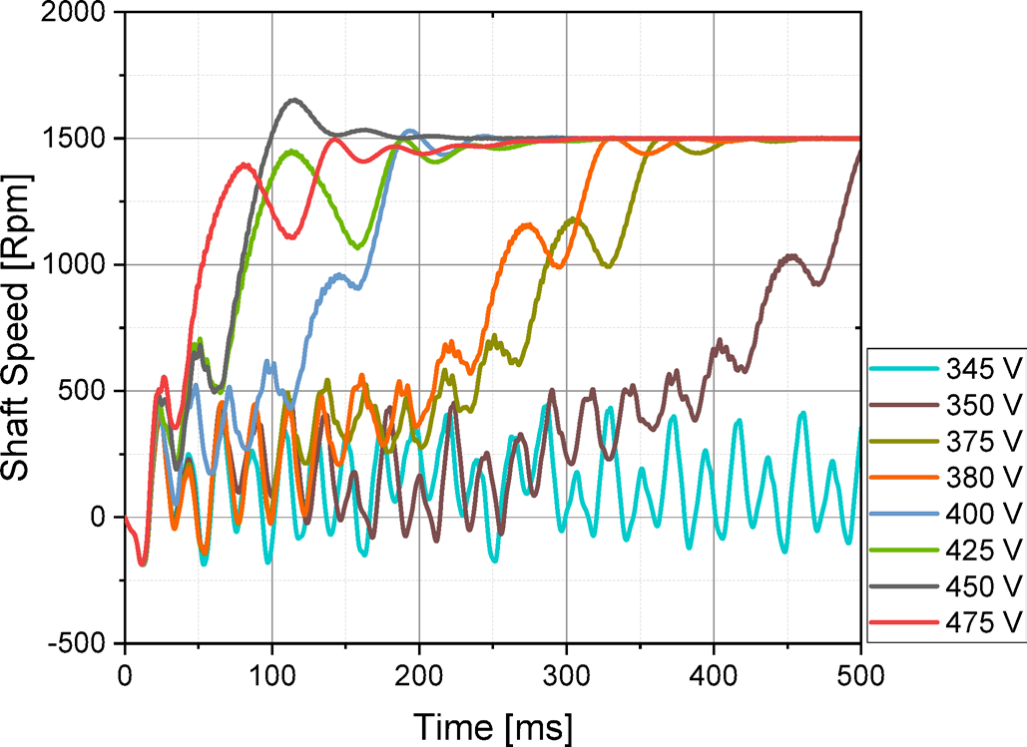
Evaluation of synchronization performance of design C at different voltage variations.

The copper rotor induction motor followed closely, along with Design C and E, which achieved fast synchronization times. Design A and D exhibited similar characteristics; however, Design A had the slowest acceleration to rated speed, showing the poorest synchronization performance among the optimized LSPMSM designs. An optimal LSPMSM is nevertheless expected to synchronize quickly, even at torque levels higher than its rated torque. For this reason, the synchronization performance of the initial and optimized candidate designs under different load conditions was analyzed. The results of these analyses are presented in Fig. 10. According to Fig. 10, the initial design could only synchronize under loads of 25 Nm or lower and failed to achieve synchronization at the rated load of 35 Nm. In contrast, the optimized designs demonstrated significantly improved synchronization performance as a result of the optimization process. Design A was only able to synchronize at its rated torque of 35 Nm but failed at higher torque levels. Design E and F successfully synchronized at 40 Nm, but failed to reach synchronous speed at 45 Nm. Design B, C, and D managed to synchronize up to 45 Nm, demonstrating better synchronization capability under higher loads. Design C achieved the shortest synchronization time at both rated torque (35 Nm) and 45 Nm, making it the fastest and most effective design. Design D had the longest synchronization time at 45 Nm, making it the least favorable among the three high-performing designs. Based on the synchronization performance analysis, Design C was identified as the optimal design, as it exhibited the fastest synchronization time at nominal load (35 Nm), the best synchronization performance at higher torque levels (45 Nm), and superior load-handling capability compared to the other candidates. Thus, Design C was selected as the optimal LSPMSM design for achieving both high efficiency and robust synchronization performance under varying load conditions. LSPMSMs are expected to maintain synchronization capability not only under overload conditions but also during voltage fluctuations. Since LSPMSMs operate directly from the mains voltage, voltage variations should not negatively impact their synchronization performance. According to the IEC 60034-28 standard, permissible voltage fluctuations in the grid are ± 5% of the nominal voltage. Therefore, the designed motor must be capable of operating at full performance within this permitted voltage range. To evaluate this, parametric simulations were conducted to analyze the synchronization performance of Design C at different voltage levels. The analysis aimed to determine the minimum voltage at which Design C can successfully synchronize under its rated load of 35 Nm. The results of these simulations are presented in Fig. 11.

The analysis results indicate the following synchronization behavior of Design C under different voltage conditions. At nominal voltage (400 V), the rotor reaches synchronous speed in 300 ms. At higher voltages (425 V and 475 V), synchronization occurs within the same duration (300 ms) as at 400 V, indicating stability in synchronization behavior. At 450 V, synchronization is achieved in a shorter time (approximately 200 ms), and the motor speed remains stable. At lower voltages, synchronization capability declines proportionally with decreasing voltage. At 380 V, the motor synchronizes in 400 ms. At 350 V, the motor fails to synchronize within 500 ms, indicating a critical voltage threshold. These results demonstrate that Design C can successfully synchronize even beyond the ± 5% voltage fluctuation limits specified in IEC 60034-28. The motor maintains stable synchronization behavior at higher voltages, while lower voltage limits its synchronization capability, eventually preventing synchronization below 350 V.

### Comparison of starting torque performances

**Fig. 12:**
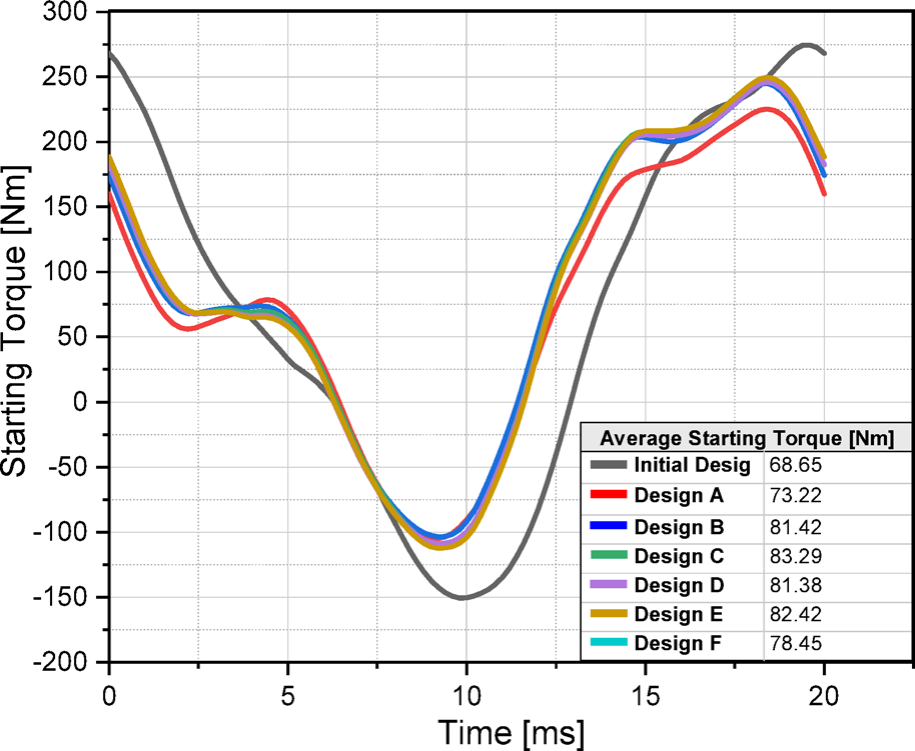
Comparison of starting torque performances.

One of the key reasons for the widespread use of induction motors in industry is their ability to start under load. Due to their hybrid rotor structure, LSPMSMs can also generate starting torque under load -similar to induction motors- once power is applied. For LSPMSMs to be a direct replacement for induction motors, they must exhibit adequate starting performance under load. To assess this, the six candidate designs were analyzed for their load-starting capabilities. In these analyses, the torque induced in the rotor over one period was examined at rated voltage while the motor speed was at 0 rpm. The comparative results of the induced torques of the initial and the six candidate designs are presented in Fig. 12.

**Fig. 13:**
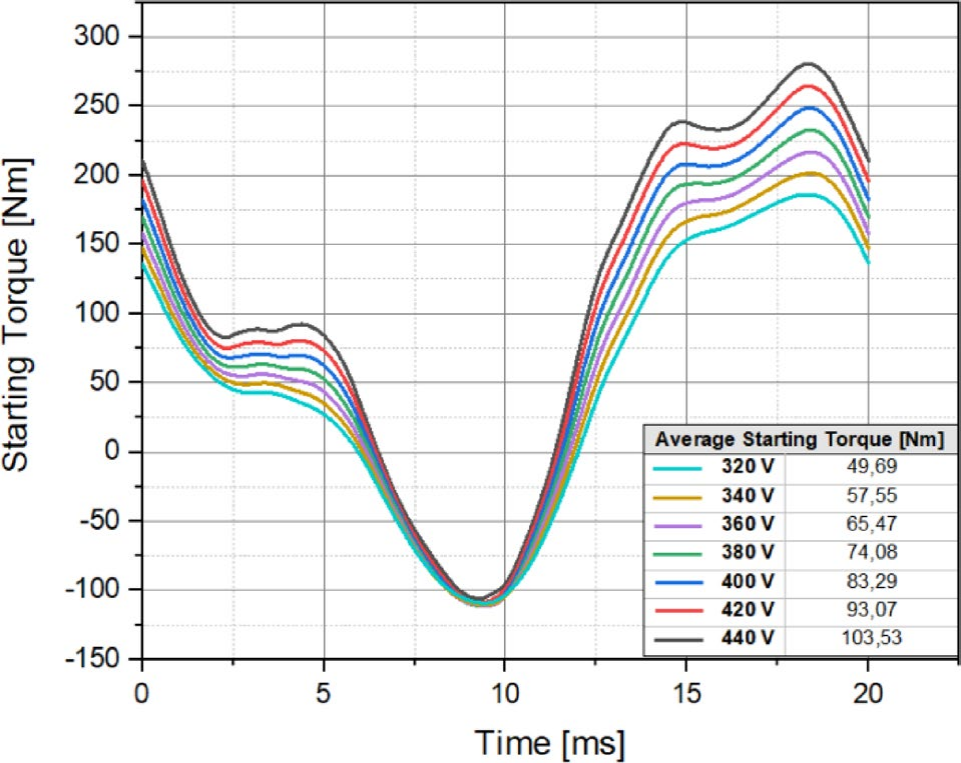
Starting performance of design C under voltage variations.

By analyzing the results in Fig. 12, the following conclusions can be drawn regarding the starting torque capabilities of the designs. The initial design exhibited the lowest starting torque performance at 68.65 Nm. However, as a result of the optimization efforts focused on improving the starting torque, all six candidate designs achieved significantly higher starting torque values. Design C produced the highest starting torque at 83.29 Nm, making it the best-performing design in terms of starting torque capability. Design A generated the lowest starting torque, producing 73.22 Nm, making it the least favorable candidate. The remaining designs achieved the following starting torque values: Design B – 81.42 Nm; Design D – 81.38 Nm; Design E – 82.42 Nm; and Design F – 78.45 Nm. These results clearly indicate that Design C provides the most effective starting torque performance, making it the optimal design for ensuring reliable starting torque capability in industrial applications where direct replacement of induction motors is required. An optimized LSPMSM is expected to exhibit high starting capability even at lower voltages. Since LSPMSMs operate directly from the mains voltage without the need for a drive, voltage drops in the grid should not significantly degrade the motor’s starting performance. To assess this, the starting torque capability of Design C was analyzed across a voltage range from 320 V to 440 V, using parametric simulations at 20 V intervals. The results of Design C’s starting torque performance under different voltage levels are presented in Fig. 13. At 320 V, Design C produced 49.69 Nm, which is above the nominal torque (35 Nm), ensuring successful startup even under low voltage conditions. At 440 V, the motor generated 103.53 Nm, demonstrating a significant increase in starting torque at higher voltages. At rated voltage (400 V), Design C achieved 83.29 Nm, which is 2.38 times the nominal torque, confirming its robust load-starting capability. These results indicate that Design C can reliably start even at lower voltages (320 V) while providing strong starting torque across the entire voltage range. This makes it a suitable replacement for induction motors in direct grid-connected applications where voltage fluctuations may occur. Based on a comprehensive comparative analysis of the analytical results, demagnetization prediction at different temperatures, synchronization performance, and starting torque capability, it was determined that Design C is the most optimal candidate among the six.

Although all six candidate designs obtained through optimization exhibited relatively similar efficiency and starting torque values, detailed finite element analyses (FEA) revealed that their synchronization capability under varying load conditions and voltage fluctuations differed significantly. These differences arise from the subtle yet critical effects of rotor geometry on the motor’s magnetic behavior.

As the rotor slots become deeper, the starting torque generally improves due to increased asymmetry and rotor resistance due to skin effect, which enhances initial torque production. However, this comes at the expense of synchronization stability, which tends to degrade due to the altered magnetic flux distribution. Similarly, the dimensions and positioning of flux barriers directly influence the air-gap flux density, impacting both starting performance and synchronization capability. The size and placement of permanent magnets within the rotor are also crucial. While increasing magnet width and thickness enhances the synchronization capability by strengthening the magnetic pull into synchronism, it also increases braking torque, which can negatively affect the motor’s ability to start under load. These competing effects illustrate the inherent trade-offs in LSPMSM rotor design, where careful balancing between synchronization stability and starting performance is essential. Among the six candidates, Design C achieved the most balanced performance due to its relatively deeper rotor slots and larger magnet volume, leading to improved starting torque and robust synchronization across a range of voltage and load variations. The magnetic topology of Design C thus provided the optimal compromise between key electromagnetic parameters, making it the most viable solution in terms of overall performance and reliability.

As a result, in the next section, FEA simulations were conducted for Design C, providing the final validation before prototype manufacturing.

### Final validation by FEA analysis

**Fig. 14:**
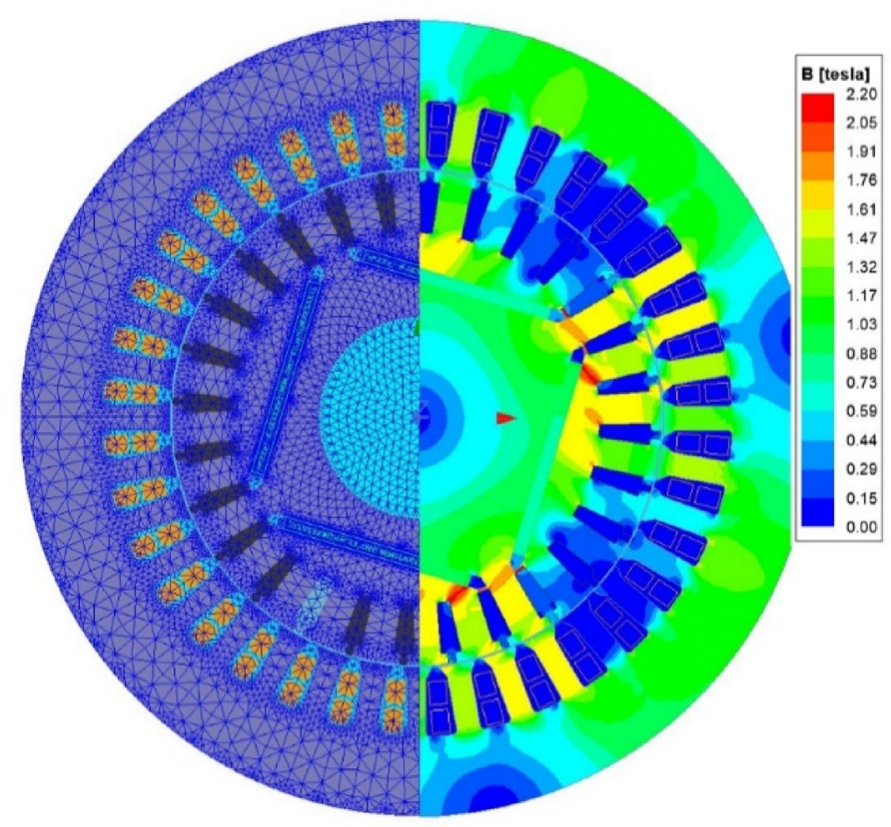
The mesh and magnetic flux density distribution of the optimal design.

Following the comparative evaluation of multiple performance parameters, the optimal design (Design C) was further analyzed using FEA in ANSYS Maxwell 2D to obtain the magnetic flux distribution, torque variation over time, and input/output power characteristics. The mesh and magnetic flux density distribution of the optimal design are given in Fig. 14.

**Fig. 15:**
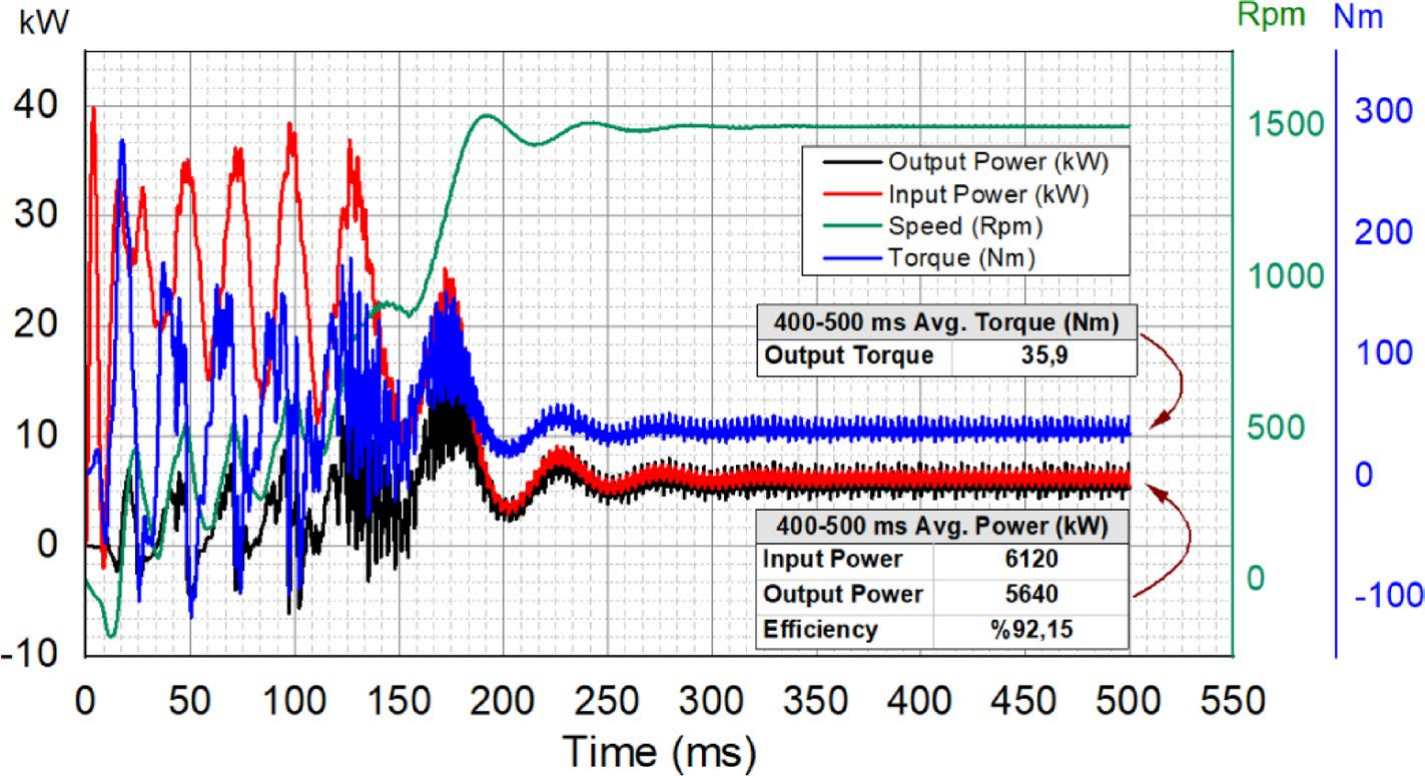
Transient analysis and efficiency validation of the optimal design.

To ensure high accuracy in the FEA analysis, a dense mesh structure was utilized in the simulations. The magnetic flux distribution was examined 400 ms after synchronization to evaluate the steady-state performance. The maximum flux density in the stator teeth was found to be 1.6 T, while in the rotor teeth it reached 1.8 T. Since the objective of this study is to design a high-efficiency motor, the input and output power under load conditions were also analyzed using transient simulations. The results of these power evaluations are presented in Fig. 15.

By examining the transient FEA analysis results presented in Fig. 15, it is observed that after overcoming initial inertia (300 ms and beyond), the motor operates at an efficiency of 92.15%. This efficiency level meets the IE4 Super Premium efficiency standard, validating the design goal. The analysis results confirm that the optimized motor successfully meets the targeted performance criteria. Based on these findings, a prototype was manufactured, and experimental tests were conducted to further validate the design.

## Fabrication and verification

Starting from a reference induction motor design, an optimization study was conducted using MOGA optimization algorithm. Among the six candidate designs obtained through optimization, a detailed evaluation was carried out based on analytical analysis, demagnetization prediction, synchronization performance, starting torque capability, voltage variations, and FEM. Following these evaluations, Design C was identified as the most optimal solution. A prototype of Design C was manufactured, tested, and subjected to a design validation process to confirm its performance and efficiency.

In the prototype, mechanical components such as the stator, frame, front and rear end bells, and cooling fan were kept unchanged to ensure compatibility with the reference motor. The manufacturing process of the optimized rotor included the following steps: The short-circuit rings were manufactured using the aluminum injection molding method. The permanent magnets were positioned in the rotor locations according to the optimized design. To keep the magnets stable in their slots, the rotor was encapsulated on both sides with a non-magnetic material. This manufacturing approach ensured that the optimized LSPMSM rotor was structurally robust and aligned with the design specifications, while maintaining compatibility with the existing motor assembly.

**Fig. 16:**
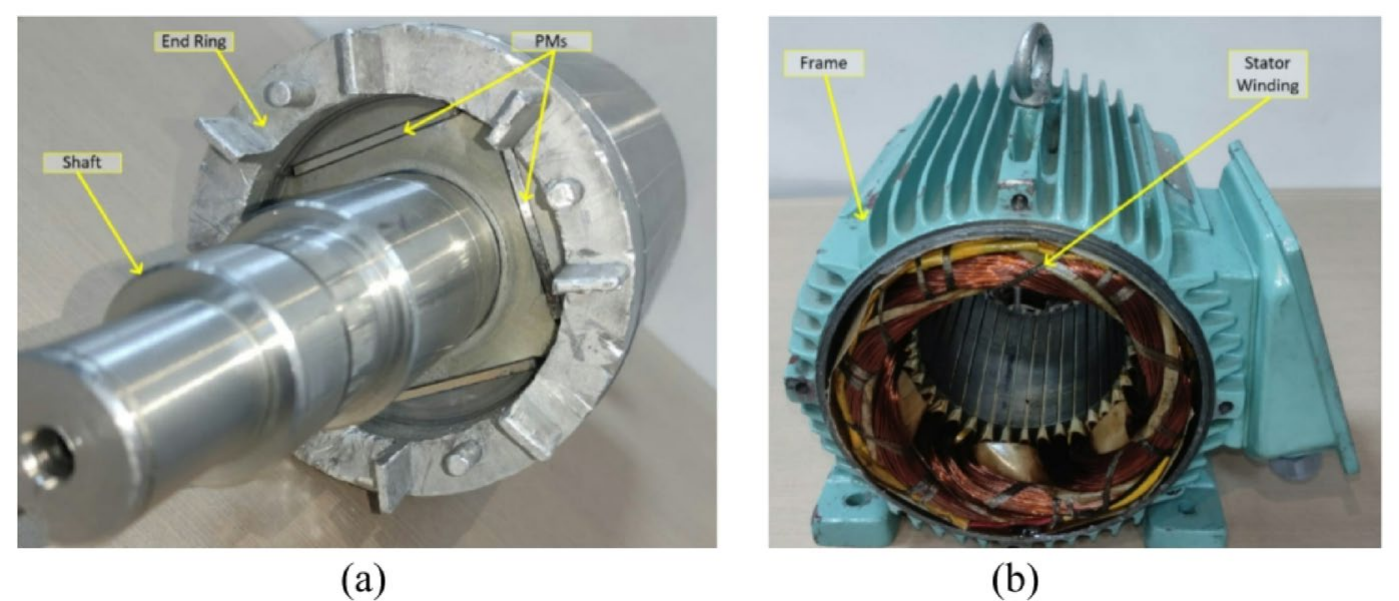
Prototyped LSPMSM (**a**) rotor, (**b**) stator.

**Fig. 17:**
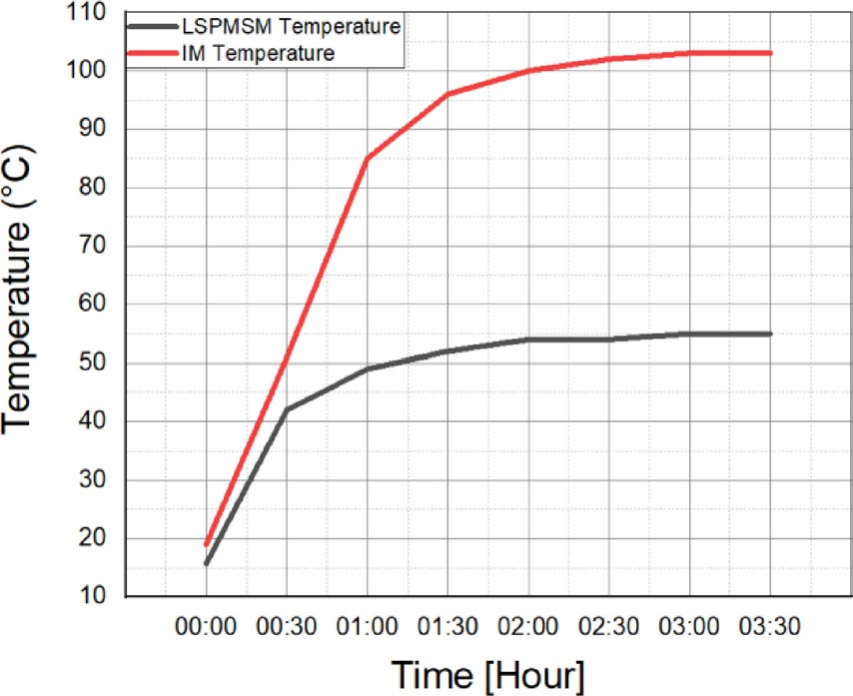
Temperature rise test of the reference IM and LSPMSM.

The manufactured LSPMSM rotor is shown in Fig. 16a, while the standard induction motor frame is presented in Fig. 16b. These images illustrate the integration of the optimized rotor into the existing motor structure, ensuring compatibility with the original stator and mechanical components. After the manufacturing process, a thermal performance test was conducted to evaluate the steady-state performance of the LSPMSM prototype. The test was performed at 400 V, 50 Hz mains voltage while the motor operated at synchronous speed. The purpose of the test was to analyze the temperature rise and thermal stability of the motor under normal operating conditions. The results of the thermal performance test provide crucial insights into the motor’s efficiency, heat dissipation, and long-term operational stability. During the thermal performance test, the motor was loaded at its rated torque. The test continued until the temperature change in the stator windings was less than 2 K in the final hour, indicating thermal stability. After confirming thermal stability, a locked-rotor test was performed to measure the starting torque generated. The same thermal and locked-rotor tests were also conducted on the reference induction motor for comparative evaluation. Temperature rise test of the reference IM and LSPMSM were analyzed and are presented comparatively in Fig. 17. These results provide insights into torque performance, starting capability, and thermal efficiency differences between the two motor types.

As specified in the IEC 60034-1 standard, the motors were tested at rated load until the temperature change in the final hour remained below 2 K. The stator winding temperature was monitored using PT100 temperature sensors embedded within the windings. Once the temperature change stabilized below 2 K, the test was terminated. To further validate the temperature rise, stator winding resistances were measured, and the temperature increase was recorded using the resistance method.

16 $$\:{T}_{rise}=\frac{{R}_{2}}{{R}_{1}}\times\:\left(T+{t}_{1}\right)-\left(T+{t}_{a}\right)$$

At the beginning and end of the test, the stator winding resistance values were recorded. Using Eq. 16, the temperature rise was calculated based on the resistance method specified in the standard and is presented in Table 7.

**Table 7:** Measurement of the temperature rises.

Motor type	Temperature point	Unit	Begin of the test	End of the test	Change in temperature rise	Measurement method
LSPMSM	Stator Winding	Ohm	1.793	2.100	40	Resistance
		°C	15.7	55	39.3	PT100 Sensor
	Ambient	°C	16.2	19.7	3.5	
Reference IM	Stator Winding	Ohm	1.66	2.15	70	Resistance
		°C	19	103	84	PT100 Sensor
	Ambient	°C	17.3	21.5	4.2	

**Fig. 18:**
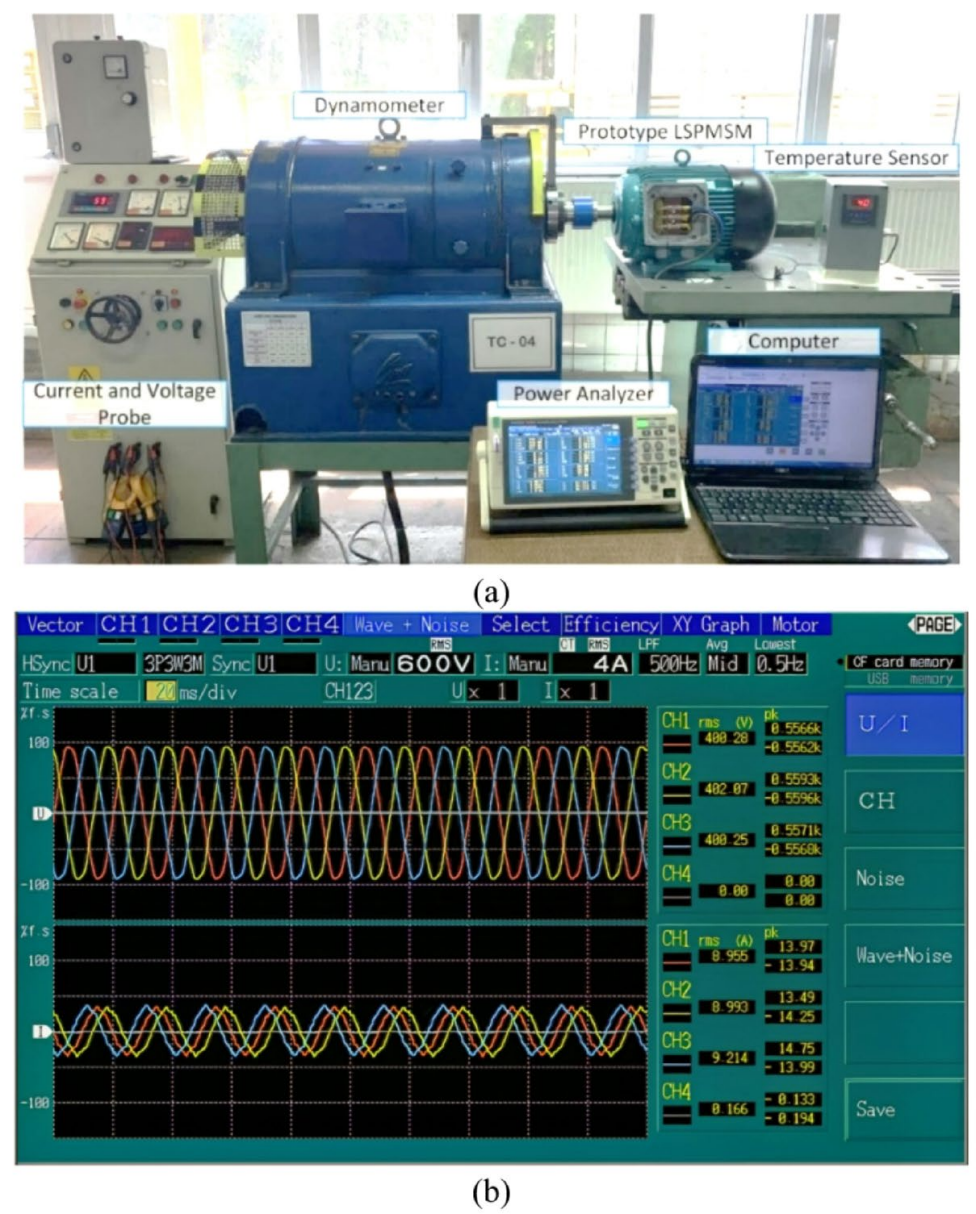
Experimental system (**a**) motor test bench and measurement devices, (**b**) voltage and current measurement by Hioki 3390.

The motor test bench and measurement devices used in the experimental study are shown in Fig. 18a. The experimental tests were performed using a DC generator-type dynamometer (Hans Still A.G. Hamburg, Model GLF 184 − 24). The dynamometer has a maximum power capacity of 7.5 kW, maximum torque of 47.75 Nm, and a maximum rotational speed of 3000 rpm. During the test, current, voltage, power factor, and corresponding power values were measured using a Hioki 3390 power analyzer and recorded in real time via a computer system for detailed analysis, as seen in Fig. 18b.

During the test, current, voltage, and power factor values were measured using a Hioki 3390 power analyzer and recorded via a computer system for detailed analysis. Following the same test procedure, the reference induction motor was also tested. The test results of the manufactured Design C were then compared with the FEM analysis results and the test data of the reference induction motor. The comparative results are presented in Table 8.

**Table 8:** Performance comparison of the reference IM and developed LSPMSM.

Item	Value					Unit
	Reference IM test result	Initial LSPMSM FEM result	Optimum LSPMSM FEM result (Design C)	Optimum LSPMSM test result	Difference (%)	
Output power	5553	5662	5640	5500	−2.5%	W
Speed	1429	1500*	1500	1500	-	rpm
Torque	36.68	36.04	35.9	35	−2.5%	Nm
Core loss	219	90.1	86.4	113.5	+ 31.36%	W
Stator copper loss	456	379.6	303.6	278	−8.44%	W
Rotor copper loss	279	-	-	-	-	W
Additional loss	33	30	30	30	-	W
Mechanical loss	60	60	60	60	-	W
Total loss	1047	559.7	480	481.5	~ 0%	W
Efficiency	84.14%	91.00%	92.15%	91.95%	~ 0%	%
Power factor	0.843	0.987	0.951	0.953	~ 0%	-
Locked rotor torque	91.7	68.65	83.29	78.75	−5.45%	Nm
Magnet weight	-	1.06	0.585	0.585	0%	kg
Change in temperature Rise (PT100 Sensor)	70	-	-	40	-	-

*Initial LSPMSM FEM results presented in this table correspond to the motor’s performance under synchronized operation. However, as shown in Fig. 9, the initial design fails to synchronize at rated load. This clearly highlights the necessity for the optimization process as undertaken in this study.

The comparison of FEM analysis results and test results in Table 8 shows minimal deviation, confirming the accuracy of the simulations. During the design and testing process, the additional loss was assumed to be 0.5% of the output power. Additionally, in the FEM analysis, the additional losses and mechanical losses of the LSPMSM were considered equal to those of the reference motor. The efficiency improved from 84.05% to 91.95%, achieving a 9.4% increase. The power factor increased from 0.843 to 0.953, marking a 13% improvement. The locked-rotor torque was calculated as 83.29 Nm in the FEM analysis, while the measured test value was 78.75 Nm, representing a 5.45% deviation. The measured 78.75 Nm starting torque is 2.25 times the rated torque, which is very close to that of the reference IM and is acceptable for LSPMSMs. These results confirm that the optimized LSPMSM meets high-efficiency and reliable performance standards, making it a suitable alternative to induction motors in industrial applications.

## Cost based evaluation of the proposed LSPMSM and reference IM

In this study, an induction motor with an IE1 efficiency level was redesigned as an IE4 efficiency level LSPMSM by modifying only the rotor structure while keeping the core length unchanged. Increasing the efficiency level leads to a reduction in energy consumption. However, although the LSPMSM offers higher efficiency, the permanent magnets used in the rotor increase the overall cost of the motor. The cost comparison between the reference IM, the proposed LSPMSM, and the rotor replacement from IM to LSPMSM is presented in Table 9.

**Table 9:** Cost of the reference IM and LSPMSM.

Part	Price
Cost of Reference IM	550 €
Cost of Proposed LSPMSM	800 €
Cost of IM to LSPMSM Rotor Replacement	300 €

In the cost analysis, the rotor cost of the reference induction motor was assumed to be 25% of the total motor cost. When the cost of permanent magnets and the labor cost for magnet placement were added to the induction motor rotor cost, the approximate total cost was estimated as €300. All subsequent calculations were based on these cost estimations.

When calculating the payback period, three different scenarios were considered for the implementation of a 5.5 kW IE4 efficiency class electric motor. In these calculations, it was assumed that the motor operates for 3000 h per year and that the electricity cost is 18.67 *€*ct/kWh^30^.

**Table 10:** Payback period cases.

Cases		Calculated payback period (year)
1	Rotor of the reference IE1 induction motor is replaced with the optimized IE4 LSPMSM rotor	0.95
2	Purchasing a New IE4 LSPMSM Motor	0.79
3	Purchasing a new IE4 LSPMSM while an IE1 motor is already in operation	2.54

The payback period was calculated based on three different scenarios for implementing a 5.5 kW IE4 efficiency class electric motor:

### Case 1

Replacing Only the Rotor: The rotor of the reference IE1 induction motor is replaced with the optimized LSPMSM rotor, while keeping the existing stator and frame.

### Case 2

Purchasing a New LSPMSM Motor: A new LSPMSM motor is purchased instead of an IE1 induction motor when investing in a new motor.

### Case 3

Replacing an Existing IE1 Induction Motor with a New LSPMSM: An existing IE1 induction motor in operation is completely replaced with a new IE4 class LSPMSM.

For each case, the payback period was calculated based on the annual energy savings and investment cost, as detailed in Table 10.

17 $$\:Energy\:Saving\:\left(kWh\right)=\left({P}_{IM\_Loss}-{P}_{LSPMSM\_Loss}\right)\text{*}{t}_{hour}$$

18 $$\:Cost\:Saving\:\left({\epsilon}\right)={P}_{Saving}\text{*}{Price}_{kWh}$$

19 $$\:Payback\:Period\:\left(Year\right)=\frac{Additional\:Cost\:for\:Efficiency\:Upgrade}{Operating\:Time\times\:kWh\:Cost}$$

**Fig. 19:**
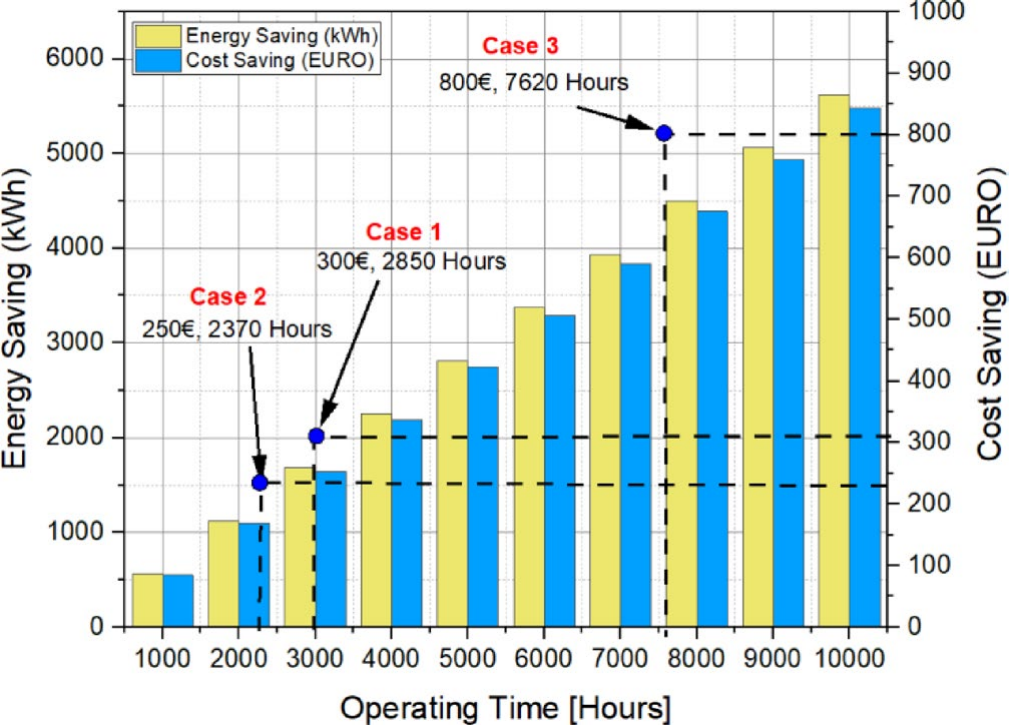
Cost comparison and payback period analysis for three scenarios.

In the cost comparison and payback period analysis, three different implementation scenarios were considered. The first scenario involves replacing only the rotor of the reference induction motor with an LSPMSM rotor. The rotor of the reference IE1 induction motor is replaced with the optimized LSPMSM rotor, while keeping the existing stator and frame. In this case, the additional investment is recovered after 2850 h of operation. The second scenario considers purchasing a new IE4 LSPMSM motor instead of an IE1 induction motor when investing in a new motor. The cost difference between the two motors was €250, and the payback period was calculated as 2370 h of operation. The third scenario involves replacing an operational IE1 induction motor with a new IE4 LSPMSM motor. The cost of a new IE4 LSPMSM motor is €800, and the payback period was found to be 7620 h, approximately 2.54 years. The variations in energy savings and cost savings based on operating hours for these three scenarios are illustrated in Fig. 19.

Energy savings were calculated by multiplying the difference between the total losses of the IE1 efficiency class induction motor and those of the LSPMSM by the motor’s operating time. Cost savings were determined by multiplying the amount of energy saved by the unit energy price, and the payback periods were calculated using the graph presented in Fig. 19. According to the analysis and test results, while this may not apply to all induction motors, it has been observed that induction motors with a high fill factor can be upgraded to the IE4 efficiency class simply by replacing the rotor.

## Conclusion

This study presented an innovative approach to upgrading an IE1 efficiency class induction motor to an IE4 efficiency class LSPMSM by optimizing the rotor structure while keeping the stator, frame, winding configuration, and mechanical components unchanged. The optimization process focused on rotor slot and magnet geometry, magnet dimensions, and core length, using MOGA to maximize efficiency with minimal cost increases. Six candidate designs were obtained and evaluated through demagnetization prediction, synchronization, and starting torque capability analyses. Design C demonstrated the most robust performance across all criteria, including stable synchronization under voltage fluctuations and the highest starting torque of 82.29 Nm, making it the optimal choice. 2D transient finite element analyses confirmed that Design C met all performance requirements. A prototype was manufactured and tested, achieving an efficiency of 92.15% in FEA analysis and 91.95% in thermal testing, thus confirming compliance with IE4 efficiency standards according to IEC 60034-30-1.

This study demonstrates that the efficiency level of an IE1 induction motor can be upgraded to IE4 by replacing only the rotor. Additionally, thermal testing showed that the LSPMSM exhibited lower temperature rise, suggesting that further efficiency gains could be realized with a lower-loss fan and an optimized housing design. Economic feasibility was assessed through three payback cases. The results showed that purchasing a new motor for a first-time investment offered the highest long-term savings, while replacing the rotor of an existing motor provided the fastest payback period of 0.95 years when an operational IE1 induction motor is already in use. The third scenario resulted in the longest payback period due to the simultaneous cost of having two motors.

While the proposed approach is experimentally validated and offers promising results, some limitations must be acknowledged. The study assumes that the stator geometry and winding configuration remain unchanged, which may restrict its direct applicability to motors with different power ratings, frame sizes, or winding topologies. Although general-purpose induction motors with IEC 132 frame size typically exhibit similar combinations of stator and rotor slot numbers, as well as comparable rotor diameters, designs with a different number of stator slots or rotor diameters may require individually optimized LSPMSM rotor configurations to achieve satisfactory performance. Furthermore, although the optimization process has improved starting torque performance, it should be noted that the presence of permanent magnets in the rotor still introduces a braking torque during motor startup. This characteristic may limit the applicability of LSPMSMs in high-inertia load applications. Therefore, such motors should primarily be considered for fan, pump, and compressor applications, where braking torque does not adversely impact startup and synchronization performance.

Despite these constraints, the methodology provides a viable pathway for improving energy efficiency in installed motor fleets, especially in industrial sectors where thousands of IE1-rated motors are still in operation. Future work may focus on generalizing this approach for different power ranges, validating its applicability to various motor series, and exploring automated retrofitting techniques. In addition, these efforts can be complemented by addressing the current constraints through the investigation of alternative rotor topologies and the implementation of advanced manufacturing techniques. In conclusion, this study provides a cost-effective solution for achieving IE4 efficiency by upgrading the rotor of existing induction motors, offering significant energy savings, a rapid payback, and strong practical potential for widespread implementation in industrial applications.

## Data Availability

The datasets used and analyzed during the current study are available from the corresponding author upon reasonable request.

## References

[1] European Directive 2005/32/EC. *A Framework for the Setting of Ecodesign Requirements for energy-using Products*L191 (Official Journal of the European Union, 2005).

[2] European Commission. *Regulation (EC) 640/2009 on Implementing Directive 2005/32/EC of the European Parliament and of the Council with Regard To Ecodesign Requirements for Electric Motors* (Official Journal of the European Union, 2009).

[3] European Commission. *Regulation (EU) 2019/1781 on Ecodesign Requirements for Electric Motors and Variable Speed Drivers* (Official Journal of the European Union, 2019).

[4] International Electrotechnical Commission (IEC). IEC 60034-30-1: Rotating electrical machines - Part 30 – 1: Efficiency classes of line operated AC motors (IE code). (2014).

[5] Kim, H. et al. A study on the rotor design of line start synchronous reluctance motor for IE4 efficiency and improving power factor. *Energies* **13** (21), 5774. 10.3390/en13215774 (2020).

[6] Kahourzade, S., Mahmoudi, A., Hew, W. P. & Uddin, M. N. Design and performance improvement of a line-start PMSM. *2013 IEEE Energy Convers. Congress Exposition*, 5042–5047 (Denver, CO, USA, 2013) 10.1109/ECCE.2013.6647381.

[7] Sorgdrager, A. J., Wang, R. J. & Grobler, A. J. Multiobjective design of a line-start PM motor using the Taguchi method. *IEEE Trans. Ind. Appl.* **54** (5), 4167–4176. 10.1109/TIA.2018.2834306 (2018).

[8] Waheed, A., Kim, B. & Cho, Y. H. Optimal design of line-start permanent magnet synchronous motor based on magnetic equivalent parameters. *J. Electr. Eng. Technol.* **15**, 2111–2119. 10.1007/s42835-020-00464-z (2020).

[9] Ocak, C. A FEM-based comparative study of the effect of rotor bar designs on the performance of squirrel cage induction motors. *Energies* **16** (16), 6047. 10.3390/en16166047 (2023).

[10] Dinh, B. M. Optimal rotor design of line start permanent magnet synchronous motor by genetic algorithm. *Adv. Sci. Technol. Eng. Syst. J.* **2** (3), 1181–1187. 10.25046/aj0203149 (2017).

[11] Behbahanifard, H. & Sadoughi, A. Cogging torque reduction in line start permanent magnet synchronous motor. *J. Electr. Eng. Technol.* **11** (4), 878–888. 10.5370/JEET.2016.11.4.878 (2016).

[12] Dinh, B. M. & Tien, H. M. Maximum efficiency design of line start permanent magnet synchronous motor. *2016 IEEE International Conference on Sustainable Energy Technologies (ICSET)*, Hanoi, Vietnam, 350–354. (2016). 10.1109/ICSET.2016.7811808

[13] Park, H. J., Hong, H. B. & Lee, K. D. A study on a design considering the transient state of a line-start permanent magnet synchronous motor satisfying the requirements of the IE4 efficiency class. *Energies* **15** (24), 9644. 10.3390/en15249644 (2022).

[14] Waheed, A. & Ro, J. Analytical modeling for optimal rotor shape to design highly efficient line-start permanent magnet synchronous motor. *IEEE Access.* **8**, 145672–145686. 10.1109/ACCESS.2020.3014718 (2020).

[15] Ghoroghchian, F., Aliabad, D., Amiri, E. & A., & Design and analysis of consequent-pole line start permanent magnet synchronous motor. *IET Electr. Power Appl.* **14** (6), 678–684. 10.1049/iet-epa.2019.0524 (2020).

[16] Palangar, M. F., Soong, W. L., Bianchi, N. & Wang, R. J. Design and optimization techniques in performance improvement of line-start permanent magnet synchronous motors: A review. *IEEE Trans. Magn.* **57** (9), 1–14. 10.1109/TMAG.2021.3098392 (2021).

[17] Baek, S. W., Kim, B. T. & Kwon, B. I. Practical optimum design based on magnetic balance and copper loss minimization for a single-phase line start PM motor. *IEEE Trans. Magn.* **47** (10), 3008–3011. 10.1109/TMAG.2011.2158077 (2011).

[18] Saha, S., Choi, G. D. & Cho, Y. H. Optimal rotor shape design of LSPM with efficiency and power factor improvement using response surface methodology. *IEEE Trans. Magn.* **51** (11), 1–4. 10.1109/TMAG.2015.2448754 (2015).26203196

[19] Jędryczka, C., Knypiński, Ł., Demenko, A. & Sykulski, J. K. Methodology for cage shape optimization of a permanent magnet synchronous motor under line start conditions. *IEEE Trans. Magn.* **54** (3), 1–4. 10.1109/TMAG.2017.2764680 (2018).

[20] Xie, Y., Li, J., Yang, Z. & Qu, C. Optimization design of line-start permanent magnet synchronous motor based on ant colony algorithm. *2014 17th International Conference on Electrical Machines and Systems (ICEMS)*, Hangzhou, China, 75–79. (2014). 10.1109/ICEMS.2014.7013440

[21] Palangar, M. F., Mahmoudi, A., Soong, W. L. & Kahourzade, S. Design optimisation of an 8-pole line-start permanent-magnet synchronous motor. *2020 2nd International Conference on Electrical, Control and Instrumentation Engineering (ICECIE)*, Kuala Lumpur, Malaysia, 1–6. (2020). 10.1109/ICECIE50279.2020.9309557

[22] Murthy, K. V. *Computer-aided Design of Electrical Machines*pp. 223–276 (BS, 2008).

[23] Yılmaz, C., Yenipınar, B., Sönmez, Y. & Ocak, C. Optimization of PMSM design parameters using update meta-heuristic algorithms. In *Artificial Intelligence and Applied Mathematics in Engineering Problems: Proceedings of the International Conference on Artificial Intelligence and Applied Mathematics in Engineering (ICAIAME 2019)* (pp. 914–934). Springer International Publishing. (2020).

[24] Gholinejad Omran, H. R., Gholamian, S. A., Hashemnia, M. N. & Hosseini, S. M. Design optimization of a five-phase IPM synchronous motor for low-speed applications. *Iran. J. Sci. Technol. Trans. Electr. Eng.* **40**, 79–91. 10.1007/s40998-016-0009-x (2016).

[25] Libert, F., Soulard, J. & Engstrom, J. Design of a 4-pole line start permanent magnet synchronous motor. *International Conference on Electrical Machines (ICEM)*. (2002), August.

[26] Stoia, D., Cernat, M., Jimoh, A. A. & Nicolae, D. V. Analytical design and analysis of line-start permanent magnet synchronous motors. *AFRICON 2009*, 1–7. (2009)., September 10.1109/AFRCON.2009.5308177

[27] Lei, G., Zhu, J., Guo, Y., Liu, C. & Ma, B. A review of design optimization methods for electrical machines. *Energies, 10*(12), 1962. (2017). 10.3390/en10121962

[28] You, Y. M. & Yoon, K. Y. Multi-objective optimization of permanent magnet synchronous motor for electric vehicle considering demagnetization. *Appl. Sci.* **11** (5), 2159. 10.3390/app11052159 (2021).

[29] ANSYS, I. N. C. *Maxwell Help: Release 2021 R1* (ANSYS Inc, 2021).

[30] Eurostat. *Electricity prices components for non-household consumers - annual data (from 2007 onwards)*. (2025). 10.2908/NRG_PC_205_C

